# FRET-guided selection of RNA 3D structures

**DOI:** 10.1093/nar/gkag147

**Published:** 2026-02-25

**Authors:** Mirko Weber, Felix Erichson, Maciej Antczak, Vanessa Schumann, Josephine Meitzner, Tomasz Zok, Fabio D Steffen, Marta Szachniuk, Richard Börner

**Affiliations:** Laserinstitut Hochschule Mittweida, University of Applied Sciences Mittweida, Technikumplatz 17, 09648 Mittweida, Germany; Laserinstitut Hochschule Mittweida, University of Applied Sciences Mittweida, Technikumplatz 17, 09648 Mittweida, Germany; Institute of Bioorganic Chemistry, Polish Academy of Sciences, Noskowskiego 12/14, 61-704 Poznan, Poland; Institute of Computing Science, Poznan University of Technology, Piotrowo 2, 60-965 Poznan, Poland; Laserinstitut Hochschule Mittweida, University of Applied Sciences Mittweida, Technikumplatz 17, 09648 Mittweida, Germany; Laserinstitut Hochschule Mittweida, University of Applied Sciences Mittweida, Technikumplatz 17, 09648 Mittweida, Germany; Institute of Computing Science, Poznan University of Technology, Piotrowo 2, 60-965 Poznan, Poland; Department of Oncology, University of Zurich, University Children’s Hospital, 8008 Zurich, Switzerland; Institute of Bioorganic Chemistry, Polish Academy of Sciences, Noskowskiego 12/14, 61-704 Poznan, Poland; Institute of Computing Science, Poznan University of Technology, Piotrowo 2, 60-965 Poznan, Poland; Laserinstitut Hochschule Mittweida, University of Applied Sciences Mittweida, Technikumplatz 17, 09648 Mittweida, Germany

## Abstract

Integrative biomolecular modeling of RNA relies on refined structural collections and accurate experimental data that reflect binding and folding behavior. However, predicting such collections remains challenging due to the rugged energy landscape and extensive conformational heterogeneity of large RNAs. To overcome these limitations, we applied a Förster resonance energy transfer (FRET)-guided strategy to identify RNA conformational states consistent with single-molecule FRET (smFRET) experiments. We predicted 3D structures of a ribosomal RNA tertiary contact comprising a GAAA tetraloop and a kissing loop using three popular RNA 3D modeling tools, namely RNAComposer, FARFAR2, and AlphaFold3, yielding a collection of candidate conformations. These models were structurally validated based on Watson–Crick base-pairing patterns and filtered using an eRMSD threshold. For each retained structure, we computed the accessible contact volume of the Cy3/Cy5 dye pair using FRETraj to predict FRET distributions. These distributions were then compared and weighted against experimental smFRET data to identify conformational states compatible with the observed FRET states. Our results demonstrate that experimental transfer efficiencies can be reproduced using *in silico* predicted RNA 3D structures. This FRET-guided workflow, combined with structural validation, lays the foundation for capturing the highly diverse conformational states characteristic of flexible RNA motifs.

## Graphical Abstract

**Graphical Abstract:**
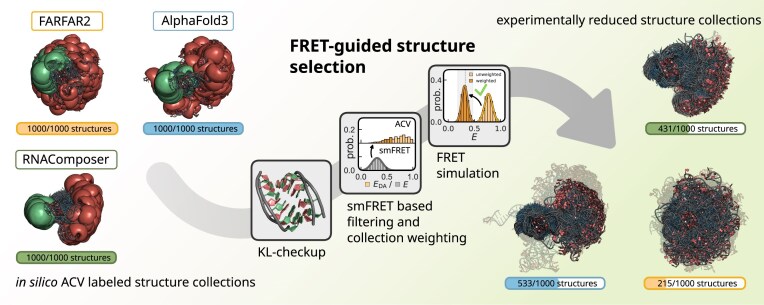

## Introduction

Three-dimensional (3D) structure prediction and molecular dynamics (MD) simulations have been instrumental in resolving biomolecular structures at atomic resolution [1]. While these methods have been particularly impactful in protein science [2–4], increasing attention is now being directed toward RNA and RNA–protein complexes due to their structural complexity and functional diversity [5, 6]. In response, RNA 3D structure prediction has become a rapidly evolving field, with sustained efforts to enhance accuracy, reliability, and robustness. A key development in this area was the launch of RNA-Puzzles in 2011 [7], a community-wide initiative for blind benchmarking of RNA 3D prediction tools against experimentally determined reference structures [8–11]. This effort has significantly accelerated the field by enabling objective performance evaluation and encouraging methodological advancements [12]. Despite a growing range of computational strategies, modeling RNA structures remains dependent on experimental data from high-resolution techniques such as X-ray crystallography [13], nuclear magnetic resonance spectroscopy [14], and cryo-electron microscopy (cryo-EM) [15]. As the demand for accurate modeling of flexible and functionally relevant RNA conformations continues to grow, the integration of predictive modeling with experimental validation remains a cornerstone of progress in RNA structural biology.

Although *in silico* prediction and MD simulations provide critical insights into the 3D organization of RNA, they often focus on identifying a single, energetically favorable state or sampling a limited subspace of the whole conformational landscape. This single-state representation is inadequate for capturing the structural heterogeneity observed in many functional RNAs, where multiple transiently populated conformations coexist [16]. To address these limitations, in-solution techniques such as small-angle X-ray scattering [17, 18], electron paramagnetic resonance (EPR) [19, 20], and Förster resonance energy transfer (FRET) [21, 22] serve as complementary approaches for probing conformationally heterogeneous RNA structure collections. These methods enable structural characterization and are particularly well suited for detecting dynamic behavior [23, 24]. EPR and single-molecule FRET (smFRET), in particular, provide access to highly precise, site-specific distance information that is critical for resolving low-populated or transient states. Within integrative or hybrid modeling approaches [25, 26], such precise distance constraints help to select and validate conformations from predicted structure collections. Among available experimental techniques, smFRET combines nanometer-scale resolution with single-molecule sensitivity, enabling direct comparison between predicted and experimental distances or entire distance distributions [27]. This makes smFRET particularly well suited for detecting conformational subpopulations underlying RNA dynamics.

RNA folding is a hierarchical, dynamic process in which proteins can assist folding by stabilizing native-like intermediates, and tertiary interactions are essential for establishing compact RNA architectures [28]. In particular, tertiary contacts between distant secondary structure elements may form transiently or only in the presence of stabilizing factors such as Mg(II) ions or RNA-binding proteins [29–32]. As a consequence, RNAs populate heterogeneous ensembles of interconverting unbound conformations rather than a single well-defined structure. Experimental characterization of such conformational collections remains challenging due to their high flexibility and structural diversity. In this context, single-molecule techniques such as smFRET identify conformations that are consistent with distance constraints measured in solution [22, 27, 33, 34].

While FRET-assisted and integrative hybrid modeling concepts are well established, the present study develops a predictor-agnostic workflow for *post-hoc* structure collection selection from *de novo* RNA 3D structure prediction outputs. Rather than fitting restraints within a single modeling engine, we combine (i) motif- and geometry-based plausibility filtering with (ii) forward modeling of smFRET efficiency distributions using explicit dye-accessibility modeling and experimentally refined photophysical parameters. This approach enables direct comparison of how different prediction algorithms populate structural diversity and how this diversity affects structure selection, consistent with FRET. Importantly, our goal is to describe solution-state heterogeneity, particularly for unbound and flexible states, rather than to infer a unique atomic structure from a single FRET observable.

Here, we apply FRET-guided integrative modeling to a ribosomal RNA model construct, featuring a kissing loop (KL) and a highly flexible GAAA tetraloop (TL_GAAA_) domain, which can serve as a tertiary contact binding to the KL [32], that we aim to characterize structurally in its unbound state. We chose this construct because it combines a structurally anchored KL motif, for which high-resolution ribosomal cryo-EM structures are available, and an unstructured region, where conformational heterogeneity is expected. This makes it a suitable test case to evaluate whether predictor-derived candidate pools can be rationally filtered and reduced to smFRET-compatible structure collections under well-defined ionic conditions. We chose three prediction algorithms, representative of distinct modeling philosophies: RNAComposer [35, 36], an efficient fragment/template-based approach; AlphaFold3 [2], an emerging deep learning-based predictor; and FARFAR2 [37], an explicit conformational sampling method. The observed differences in ensemble breadth, therefore, primarily reflect design goals rather than a nongeneralizable superiority ranking: FARFAR2, as a sampling-oriented method, employs a stepwise Monte Carlo sampling procedure guided by a scoring function, which inherently generates structurally diverse collections, albeit at the expense of high computational cost, whereas RNAComposer and AlphaFold3, as efficiency-oriented predictors, are designed for high efficiency and do not explicitly perform conformational sampling; instead, they typically return a small set of high-confidence/low-energy solutions rather than an explicitly sampled ensemble [11, 27]. For model validation, we calculated the multiple accessible contact volume (mACV) [27, 38, 39] to predict *in silico* FRET from the dye-labeled RNA models and used these to identify an initial pool of candidate structures matching the experimental smFRET distribution. To ensure structural plausibility, we applied established validation metrics, including Watson–Crick (WC) base-pairing [29] analysis and the eRMSD score [40, 41], yielding a refined collection suitable for smFRET-guided conformer selection.

Ultimately, we used the experimentally measured smFRET distribution to guide structure selection from the validated collections. Instead of comparing FRET values *post hoc*, we applied the distribution as a filter to extract conformers whose ACV-based predictions matched the experimental data. By enabling targeted sampling within the conformational landscape resolved by smFRET, this approach advances RNA structural modeling beyond static representations toward a dynamic, experimentally grounded collection.

## Methods

### Probing the unbound state with smFRET

**Figure 1.**
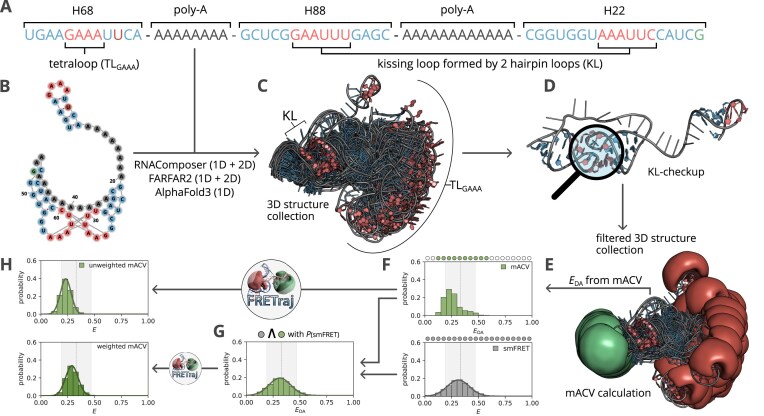
Workflow for selecting smFRET-consistent RNA 3D structure collections. The pipeline starts from a sequence and yields a final collection of 3D structures that best represent the experimental smFRET data under the condition of a correctly folded KL motif. (**A**) RNA sequence of the investigated model construct, highlighting all structural elements. (**B**) Secondary structure representation of the construct. (**C**) Initial structure collection of 1000 RNA 3D models predicted by RNAComposer. (**D**) Evaluation of predicted structures using Barnaba to ensure correctly folded KLs via WC base pairing and the calculation of the eRMSD relative to the reference structure. (**E**) For each filtered structure, the mACVs of the dyes Cy3 and Cy5 were predicted, and the mean FRET efficiency was calculated. (**F**) Distribution of the $E_{\mathrm{DA}}$ values calculated from the mACVs, shown alongside the experimental smFRET distribution with corresponding bin populations. (**G**) Reweighting of the structure collection by sampling structures from each populated bin according to the probabilities observed in the experimental smFRET distribution. (**H**) Comparison of FRET efficiencies after photon sampling with FRETraj for the unweighted and weighted $E_{\mathrm{DA}}$ distributions.

smFRET measurements of the Cy3/Cy5-labeled ribosomal RNA model construct, designed to probe KL formation and potential TL_GAAA_ binding, were performed and analyzed according to standard protocols [23, 27] in a home-built fluorescence microscope. The construct was prepared as described in [32] and visualized in Fig. 1A and B. To allow for molecular sorting, pulsed overlaid excitation (POE) smFRET experiments were performed in standard buffer containing [K$^{+}$] = $116 \,\mathrm{mmol}\,\mathrm{l}^{-1}$ and ethylenediaminetetraacetic acid to allow KL formation without TL_GAAA_ binding (Supplementary Figs S1 and S2). The smFRET data were corrected for background and crosstalk, including bleed-through and direct excitation. A $\gamma$-correction was applied to account for differences in detection efficiency and quantum yield of the FRET dye pair (Supplementary Table S1). These corrections are essential for the comparability of the experimental data with FRET distributions derived from *in silico* structure collections using FRETraj [39], together with the experimental burst size distribution (Supplementary Fig. S7B), i.e. the sum of donor and acceptor intensities (Fig. 1F). Additionally, the fluorescence lifetime of the Cy3/Cy5-labeled ribosomal RNA model construct was measured by TCSPC using a FluoTime 250 and time-resolved fluorescence anisotropy decays were calculated to investigate dye–RNA stacking (Supplementary Figs S3 and S4, and Supplementary Tables S2 and S3) to calculate the CV fraction according to [27, 38].

### *In silico* RNA 3D structure prediction and MD simulation

Structure collections consisting of 10 000 structures each were predicted using RNAComposer, FARFAR2, and AlphaFold3 (Supplementary Methods). Additionally, we performed six independent $1 \,{\mu }\mathrm{s}$ MD simulations with different initial seed structures chosen to represent structurally diverse conformations (Supplementary Methods). An equal number of structures was sampled from each of the six MD simulations for comparison with the 3D prediction tools and for subsequent use in the FRET-guided reweighting. ACVs of both dyes were calculated for each structure predicted by the 3D prediction tools, as well as every $100 \,\mathrm{p}\mathrm{s}$ along the MD trajectories. Photon emission events were simulated by FRETraj [39, 42, 43]. The FRET distributions were also analyzed both individually for each MD simulation (Supplementary Figs S6 and S7) and as a combined collection (Fig. 4A and B—MD simulation). All MD simulations were annotated every $100 \,\mathrm{p}\mathrm{s}$ based on WC base pairing using the Barnaba toolbox [41], resulting in six sets of 10 000 annotated structures representing the unbound RNA conformation (averaged in Fig. 3A right).

### Estimating structure collection size using Kullback–Leibler divergence

**Figure 2.**
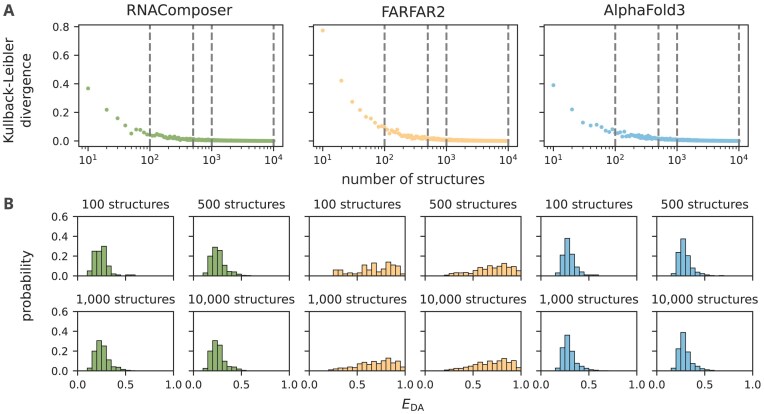
Distribution of energy-transfer efficiencies $E_{\mathrm{DA}}$ calculated from ACV-derived mean donor–acceptor distances. Distributions are shown for RNAComposer, FARFAR2, and AlphaFold3, and compared to the corresponding reference distributions obtained from the full structure collections ($N = 10\,000$ structures). (**A**) KLD of randomly sampled subsets ranging from 10 to the corresponding full set of 10 000 structures for each predictive tool. Dashed vertical lines indicate the subset sizes $i = 100, 500, 1000, \textrm {and} \,10\,000$ used to generate the $E_{\mathrm{DA}}$ distributions shown in (**B**).

The foundation of our approach to select diverse structures that match the experimental distribution of energy-transfer efficiencies is the diversity of the underlying generated 3D structure collection. Our underlying assumption is that $N = 10\,000$ predicted structures are sufficient to adequately cover the conformational search space sampled by the prediction tools considered. To assess the minimum number of structures required for a representative subset, and thus to limit the computational costs to a minimum, we compared the probability distribution $ P$ of the energy-transfer efficiencies $E_{\mathrm{DA}}$ calculated from the initial structure collection of size $N$ and sub-distributions sampled $ Q_{x_i}$ from each prediction tool. This comparison allowed us to assess how well smaller subsets capture the variability of the energy-transfer efficiency $E_{\mathrm{DA}}$ observed in the collection of structures in the particular distribution. For each $ i$, where $ i \in \lbrace 10, 20, 30, \dots , 9990\rbrace $, $ x_i$ represents the number of samples drawn from the structure collection of size $ N$ and for each $ x_i$ the structures were randomly sampled from $N$. To compare the probability distribution, we applied the Kullback–Leibler divergence (KLD) [44], which is computed for each repetition as:


\begin{eqnarray*}
D_{\mathrm{KLD}}(P \Vert Q_{x_i}^{(r)}) = \sum _{j=1}^m p_j \log \left(\frac{p_j}{q_j^{(r)}}\right),
\end{eqnarray*}

where $ m$ is the total number of bins, $ p_j$ is the true probability of bin $ j$, and $ q_j^{(r)}$ is the corresponding probability of the bin from the sampled distribution. The mean KLD across all repetitions for a given $ x_i$ is then defined as:


\begin{eqnarray*}
\overline{D_{\mathrm{KLD}}}(x_i) = \frac{1}{k_i} \sum _{r=1}^{k_i} D_{\mathrm{KLD}}(P \Vert Q_{x_i}^{(r)}),
\end{eqnarray*}

where $ k_i$ was increased until the standard deviation of the corresponding KLD values fell below 1% of their mean. Using this criterion, we determined the minimum number of structures required for each structure collection to achieve an accurate representation (Fig. 2 and Supplementary Fig. S5 for different bin sizes $\Delta E_{\mathrm{DA}}$).

### Validating KL formation and filtering structure collections

**Figure 3.**
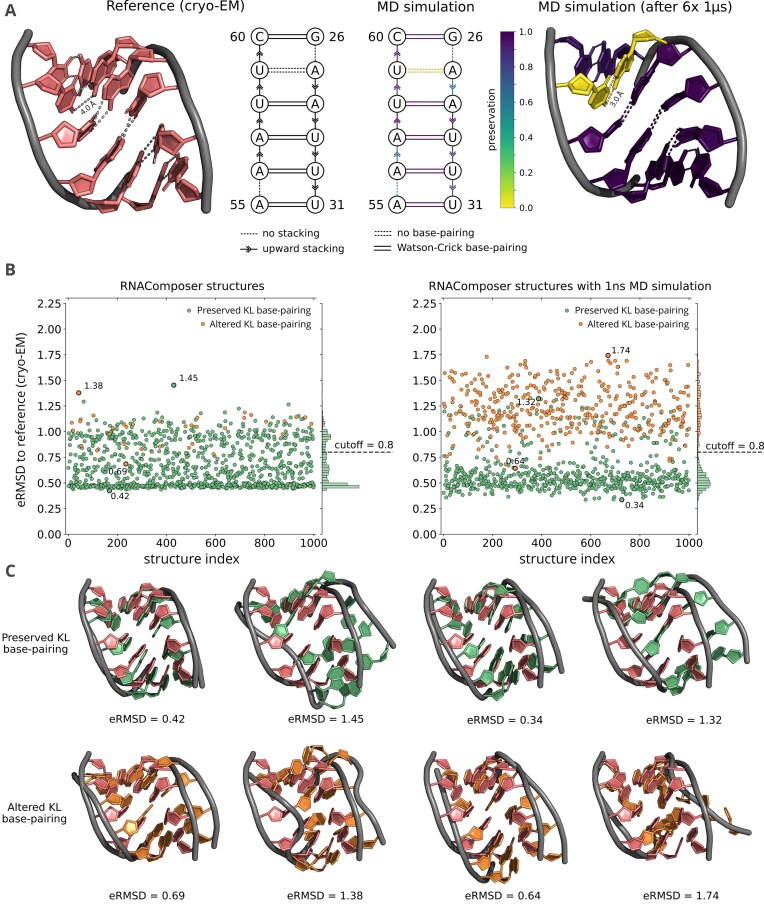
Reference KL fold and its comparison with the RNAComposer structure collection. (**A**) (left) Cryo-EM reference structure of the KL, comprising WC base pairs, except A27–U59. (right) Preservation of the KL motif across six $1 \,{\mu }\mathrm{s}$ MD simulation sets relative to the cryo-EM reference structure comprising WC base pairs for the complete KL. (**B**) eRMSD of all RNAComposer structures (left) and RNAComposer structure collection after $1 \,\mathrm{n}\mathrm{s}$ MD refinement (right) compared to the reference cryo-EM structure, focusing on the KL region. (**C**) Representative structures classified as preserved or altered KLs, showing those with the lowest and highest eRMSD, both before and after MD refinement.

To ensure formation of the KL as observed in the reference structure (PDB ID: 3JCT and Fig. 3A), we compared the WC base pairs annotated by Barnaba [41] across all predicted structures with those from 10 000 MD-derived states. This analysis validated the structural preservation of the KL motif. WC base pairs were then annotated for all structures from RNAComposer (Fig. 3A and B), FARFAR2 (Supplementary Fig. S8), and AlphaFold3 (Supplementary Fig. S9). Structures were classified as preserved KL if all relevant base pairs were canonical, except for U27–A59, which was allowed to vary in agreement with the reference. Any deviation was annotated as altered KL (Fig. 3B and C for RNAComposer and Supplementary Figs S8 and S9 for FARFAR2 and AlphaFold3).

Structural similarity of each model to the reference was quantified using eRMSD [40]. Models with $\mathrm{eRMSD} \le 0.8$ and preserved base pairing were selected for further analysis (Fig. 3B left for RNAComposer). To assess structural stability and confirm base-pair preservation after refinement, each filtered model underwent a short $1 \,\mathrm{n}\mathrm{s}$ MD simulation, followed by re-annotation and recomputation of WC base pairs and eRMSD (Fig. 3B right for RNAComposer, and Supplementary Figs S8 and S9 for FARFAR2 and AlphaFold3, respectively).

### Reweighting FRET efficiencies from mACVs

To compare the structure collections obtained from the investigated 3D prediction tools and the described MD simulations with the experimental FRET distribution, we applied FRETraj within the FRET-assisted modeling pipeline [45] to generate a distribution of energy-transfer efficiencies $ E_{\mathrm{DA}}$ based on the ACV dye model and using two different structure sampling strategies (compare Fig. 1E–H) [39]. In the first approach, each structure was selected exactly once, thereby preserving the original distribution of predicted energy-transfer efficiencies $ E_{\mathrm{DA}}$. Consequently, the relative frequency of structures in each bin $ B_i$ reflected the number of structures falling within the corresponding $ E_{\mathrm{DA}}$ range, i.e.


\begin{eqnarray*}
P_{\mathrm{unweighted}}(i) = \frac{|B_i|}{N},
\end{eqnarray*}

where $ |B_i|$ denoted the number of structures in bin $ i$, and $ N$ was the total number of structures in the filtered collection (Fig. 1F and H—unweighted mACV).

In the second approach, structures were sampled such that the resulting distribution approximated the experimental smFRET distribution $ p^{\mathrm{smFRET}}$ (Fig. 1F–H—weighted mACV):


\begin{eqnarray*}
P_{\mathrm{weighted}}(i) = \left\lbrace \begin{array}{@{}l@{\quad }l@{}}p_i^{\mathrm{smFRET}} & \text{if } |B_i| > 0, \\0 & \mathrm{otherwise,} \end{array}\right.
\end{eqnarray*}

where $ p_i^{\mathrm{smFRET}}$ denoted the smFRET probability of bin $ i$, provided that $ B_i$ was not empty.

Based on this, two scenarios were distinguished for each bin:

#### Scenario 1:

If the relative number of structures in $ B_i$ exceeded the experimental probability $ p_i^{\mathrm{smFRET}}$, a subset of structures was randomly sampled from $ B_i$ to match $ p_i^{\mathrm{smFRET}}$.

#### Scenario 2:

If the number of structures in $ B_i$ was insufficient to satisfy $ p_i^{\mathrm{smFRET}}$, all structures in $ B_i$ were selected repeatedly until the required number was reached. Any remaining fraction was filled by randomly sampling additional structures from $ B_i$. Each repetition was randomly permuted to construct an expanded set $ \bar{B}_i$, from which sampling was performed to match $ p_i^{\mathrm{smFRET}}$.

## Results

### One thousand RNA 3D structures of the KL-TL_GAAA_ are sufficient to capture experimental FRET distributions

*In silico* structure prediction methods generally struggle to identify how many distinct structures are required to represent a structurally dynamic state. Thus, a collection of structures in one conformation is sampled from a diverse set of static structures.

In our work, we compared three widely used tools, RNAComposer, FARFAR2, and AlphaFold3, and evaluated their ability to predict the unbound state of the KL-TL_GAAA_ model construct. Each tool generated 10 000 structures, serving as a baseline for assessing the structural diversity of the unbound state (Fig. 2). Subsets of varying sizes were then sampled from each baseline dataset and compared to the full $E_{\mathrm{DA}}$ distribution using the KLD.

In this context, the bin size of the FRET distribution was crucial: the more fine-grained we represented the FRET distribution, the more structures were required to accurately represent the baseline of the full structure collection of size $N$ (Supplementary Fig. S5). A bin size of $\Delta E_{\mathrm{DA}} = 0.05$ was chosen based on a benchmark study demonstrating an experimental accuracy of $\Delta$E = $\pm$0.05 [23], and this criterion is applied throughout the work. In fact, the KLD converged toward zero across all tested $E_{\mathrm{DA}}$ bin sizes (Supplementary Fig. S5). The minimum feasible subset is determined by the diversity of the original $E_{\mathrm{DA}}$ distribution. As a result, FARFAR2 requires more structures than RNAComposer and AlphaFold3 to reproduce the baseline (Fig. 2A). RNAComposer and AlphaFold3 begin to converge at a subset size of around 100 structures, while FARFAR2 requires a slightly larger subset due to a wider range of predicted $E_{\mathrm{DA}}$ values (Fig. 2). To ensure a sufficiently large set of structures for subsequent validation while retaining enough conformers to represent the FRET distribution, we chose a structure collection size of 1000 for each tool. This number is sufficient to model FRET in the unbound (low-FRET) state of the investigated construct, although it may need adjustment for RNAs with greater structural complexity.

### Accurate structure annotation is critical for selecting reliable KLs

Since proper folding of the tertiary contact in our construct depends on an accurately positioned KL, we specifically analyzed our structure collections to ensure KL formation matched the cryo-EM reference (PDB ID: 3JCT). Folding was first assessed via WC base-pairing patterns observed in the reference structure [46]. Our analysis revealed that the hydrogen-bonding distance between A27 and U59 in the reference exceeded the favorable range for stable WC base pairing and therefore was not classified as canonical. To investigate the stability and variability of the motif, we used all six $1 \,{\mu }\mathrm{s}$ MD simulations of our model construct (Fig. 3A). The MD simulation seed structures are made up from the cryo-EM reference structure, thus starting with A27–U59 without canonical WC geometry. These simulations show that all base pairings, including A27–U59, adopt canonical WC geometry, indicating improved consistency in the MD collection. For subsequent classifications of the KL integrity, we required canonical WC base pairing for all bases except A27–U59, for which spatially proximal representations not necessarily classified under a specific WC base-pairing category were allowed.

To complement WC base-pairing analysis and refine structural characterization of the KL, we computed the eRMSD for all predicted structures relative to the cryo-EM reference (PDB ID: 3JCT). The eRMSD captures geometric deviations in base pairs and stacking interactions, providing a robust measure of RNA motif similarity. Across prediction tools, many structures clustered around an eRMSD $\approx$ 0.5 (Fig. 3B left and Supplementary Figs S8 and S9 left). RNAComposer and FARFAR2 showed gradual transitions to higher eRMSD, whereas AlphaFold3 exhibited a distinct cutoff at $\mathrm{eRMSD} = 0.8$, and we apply this value for the assessment of all predictive tools. To assess structural plasticity, each structure was subjected to a short $1 \,\mathrm{n}\mathrm{s}$ MD simulation, followed by re-annotation of base pairings and re-calculation of eRMSD (Fig. 3B right and Supplementary Figs S8 and S9 right). These simulations introduced local fluctuations in the KL, shifting some conformers above $\mathrm{eRMSD}~=~0.8$ and increasing the number of non-WC base pairs, which were dismissed for further FRET predictions.

Neither WC base pairing nor eRMSD alone suffices to verify KL preservation in our construct. Using only $\mathrm{eRMSD} \le 0.8$ can retain structures that deviate from the canonical WC pattern and thus do not represent a preserved KL. Conversely, relying solely on WC base pairing can include structures with significantly altered backbone geometries. Applying both criteria together consistently selects folded KL structures with WC base pairing across all three structure prediction tools, further used for FRET predictions (Fig. 3C and Supplementary Figs S8 and S9).

### The experimental smFRET distribution guides the selection of structures from the predicted collections

**Figure 4.**
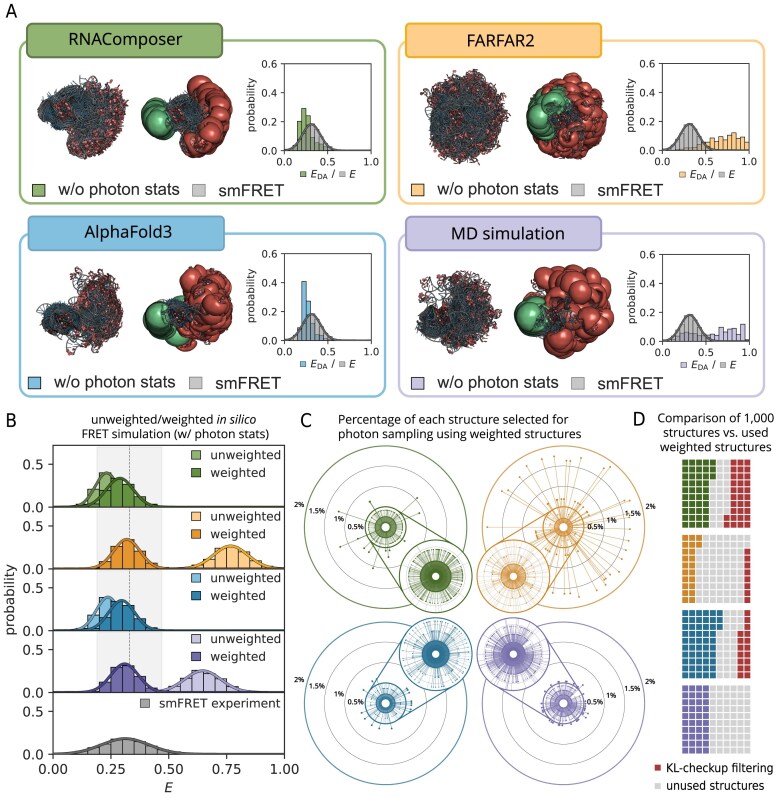
Comparison of 3D structure prediction tools and MD simulations with the smFRET experiment. (**A**) Energy-transfer efficiency $E_{\mathrm{DA}}$ distributions for all approaches without photon sampling, and the final 3D structure collections after filtering presented with and without ACVs. (**B**) FRET distributions with photon statistics simulated by FRETraj. “Unweighted” refers to a uniform contribution of each structure to the burst calculation, whereas “weighted” denotes sampling according to experimental smFRET probabilities. (**C**) The radar charts show the contribution of individual FRET-selected structures to the weighted collections for each tool. The FARFAR2 collection includes many diverse structures differing in energy-transfer efficiency, while RNAComposer and AlphaFold3 provide the most similar structures, comprising a narrow energy-transfer efficiency distribution. The FRET-guided selection leads to a fewer number of individual structures of the FARFAR2 collection, which are highly populated, while the number of structures of RNAComposer and AlphaFold3 is comparably high but uniformly distributed in the weighted FRET distribution. The population of structures from MD simulations is comparable to the FARFAR2 collection, but comprises more structures to be selected throughout the weighting process with a corresponding lower percentage in the weighted FRET distribution. (**D**) Number of structures lost during KL validation and the final number selected for weighting. KL filtering was not applied to MD simulation structures.

Building on the filtered structure collections, we can probe their conformational landscape and guide further refinement using the experimental smFRET distribution. The initial energy-transfer efficiency distributions $E_{\mathrm{DA}}$ derived from ACVs without photon sampling of all filtered structures reveal distinct conformational profiles across the different prediction strategies (Fig. 4A). FARFAR2 and the combined six MD simulations sample a broad range of inter-dye distances, covering not only the experimentally observed FRET distribution of the model construct, but higher energy-transfer efficiencies yielding mean energy-transfer efficiencies of $0.69 \pm 0.19$ and $0.60 \pm 0.24$, respectively (Table 1—w/o photon sampling). This is also evident in the structural visualizations, which show a nearly globular distribution of different TL_GAAA_ positions. In contrast, RNAComposer and AlphaFold3 predominantly yield conformations in the low-FRET region, with mean energy-transfer efficiencies of $0.26 \pm 0.08$ and $0.27 \pm 0.08$, respectively, suggesting structurally constrained collections that arise from a stretched poly(A)-linker conformation limiting the accessible space of the TL_GAAA_ (Fig. 4A and Table 1—w/o photon sampling). Interestingly, these initial distributions already indicate that none of the tools fully cover the experimentally observed FRET distribution.

**Table 1.** FRET measurements for all approaches

	w/o photon sampling	w/ photon sampling	
		Unweighted	Weighted
RNAComposer	0.26 $\pm$ 0.08	0.23 $\pm$ 0.05	0.29 $\pm$ 0.06
FARFAR2	0.69 $\pm$ 0.19	0.77 $\pm$ 0.07	0.32 $\pm$ 0.06
AlphaFold3	0.27 $\pm$ 0.08	0.24 $\pm$ 0.05	0.30 $\pm$ 0.06
MD simulation	0.60 $\pm$ 0.24	0.65 $\pm$ 0.08	0.31 $\pm$ 0.06
smFRET	–	0.32 $\pm$ 0.14	

To directly compare our experimental smFRET distribution with the predicted structure collections, we performed photon burst simulations for each structure collection, including the three RNA prediction tools and MD trajectories. This approach incorporates experimental conditions such as shot noise broadening, direct excitation, and gamma correction of the underlying energy-transfer efficiency $E_{\mathrm{DA}}$ distribution, resulting in an *in silico* FRET distribution (Fig. 4B). We note that accurate forward modeling of FRET distributions requires experimental control of dye photophysics in the given labeling context and the ACV dye model. Here, fluorescence dynamic anisotropy measurements indicated pronounced dye–RNA interactions for the donor (Supplementary Fig. S3 and Supplementary Tables S2 and S3), consistent with a comparably high donor quantum yield (0.46) used for both FRET prediction and experimental correction. Assuming fast dynamics of the RNA model construct in the FRET experiment, bursts were averaged across MD trajectories and subsamples of predicted structures, respectively. This uniform (unweighted) sampling yields *in silico* mean FRET efficiencies of $0.23 \pm 0.05$ (RNAComposer), $0.77 \pm 0.07$ (FARFAR2), $0.24 \pm 0.05$ (AlphaFold3), and $0.65 \pm 0.08$ (MD simulation), which remain broadly consistent with the corresponding means of the underlying $E_{\mathrm{DA}}$ distributions. The resulting FRET histograms are thus uniformly sampled from all structures (Fig. 4B—unweighted and Table 1—w/ photon sampling unweighted). Notably, despite partial overlap, none of the *in silico* FRET distributions generated from RNA 3D prediction tools or MD simulations fully recapitulate the experimental smFRET distribution.

Therefore, we investigated an alternative sampling approach in which structures are not uniformly sampled, but instead selected according to their probability in the experimental smFRET distribution. This strategy enables us to restrict the selection to structures that satisfy the KL filtering criteria while simultaneously enriching conformations that are most likely to be observed in an smFRET experiment. The resulting weighted *in silico* FRET distributions (Fig. 4B—weighted) now span the experimental smFRET range across all prediction tools and the MD collection, yielding similar mean FRET efficiencies of $0.29 \pm 0.06$ (RNAComposer), $0.32 \pm 0.06$ (FARFAR2), $0.30 \pm 0.06$ (AlphaFold3), and $0.31 \pm 0.06$ (MD simulation) (Table 1—w/ photon sampling weighted). However, the experimental width of the distribution could not be fully reproduced, despite accounting for shot-noise broadening using the experimental burst-size distribution and applying gamma correction.

Notably, this probability-based selection can overrepresent individual structures when the underlying structure collections exhibit only a limited overlap with the experimental smFRET distribution. In the weighted RNAComposer collection, individual structures contribute >1% of the total collection, whereas FARFAR2 shows even higher values with single structures exceeding 1.5% and approaching 2%. In contrast, AlphaFold3 and the MD collection remain more diverse, with all individual structures contributing <1% (Fig. 4C). How strongly individual structures are overrepresented depends on the initial overlap between each KL filtered structure collection and the experimental smFRET distribution. A broader coverage provides more candidates per bin and supports more heterogeneous sampling. Consequently, the number of unique structures retained in the weighted collections differs across the prediction tools and the MD simulations. Consistent with this, the weighted structure collections comprise 431 (RNAComposer), 215 (FARFAR2), 533 (AlphaFold3), and 400 (MD) unique structures (Fig. 4D and Supplementary Fig. S11).

## Discussion

FRET-guided integrative modeling has often relied on MD simulations to generate structure collections for comparison with experimental data [25, 27, 47]. While MD provides physically grounded structure collections, it is computationally demanding and often limited in its ability to sample broad conformational landscapes, particularly for flexible RNAs, efficiently. Building on earlier work in FRET-assisted modeling [27, 48, 49], we examine whether modern RNA 3D structure prediction tools can serve as practical alternatives to MD-based sampling. A central challenge in this context is that a single smFRET distance distribution is intrinsically underdetermined for complex RNA 3D folds: even after applying physical plausibility filters, multiple conformations can remain compatible with the same observable. Accordingly, rather than interpreting the modeling outcome as a single structure, our goal is to obtain a physically plausible collection of structures that is consistent with the measured FRET distribution under defined biochemical conditions. Within this framework, we assess the number of structures required to adequately represent a heterogeneous FRET state observed in a single FRET experiment, as well as the diversity and reliability of the resulting *in silico* FRET predictions and structure collections. For unbound and conformationally flexible RNAs, retaining heterogeneous solutions is therefore not a limitation of the approach, but a realistic representation of the expected in-solution state landscape.

Most notably, our work demonstrates that RNAComposer and AlphaFold3 predominantly model the unbound state between a GAAA tetraloop and a KL receptor as an extended conformation, resulting in a narrow distribution of low energy-transfer efficiencies that overlap with the experimental smFRET data. In our construct, a poly(A) linker connects the TL_GAAA_ with the KL and the linker is not expected to form stable interactions with either element in the presence of potassium(I) only [32]. The observed dye distance $R_{\mathrm{DA}}$, and accordingly the energy-transfer efficiency, are influenced by the conformational flexibility of the linker [50–53]. Poly(A)-linkers are known to adopt stacked base conformations in transiently stable states [54, 55], which may restrict the TL_GAAA_ range of motion, consistent with conformations predicted by RNAComposer and AlphaFold3.

Interestingly, FARFAR2 generates a more diverse structure collection yielding *in silico* energy-transfer efficiencies that overlap with the experimental smFRET distribution. This includes poly(A)-linker conformations that allow greater spatial freedom of the TL_GAAA_, resulting in higher FRET efficiencies. These observations indicate that, despite identical initial conditions, the poly(A)-linker can explore a vast conformational space on the nanosecond timescale (Supplementary Figs S10 and S11).

Importantly, the differences in structural diversity observed across the three RNA 3D prediction approaches should not be interpreted in terms of “superior” or “inferior” performance, but rather as a consequence of their distinct design philosophies within the RNA-Puzzles community [11]. FARFAR2 is explicitly designed to sample the conformational space via fragment- and base-step sampling, combined with a physics-inspired scoring function, thereby generating structure collections that populate multiple low-energy basins. While this strategy enables broad structural diversity, it incurs substantial computational cost, and exhaustive sampling remains challenging for larger RNAs [37]. In contrast, RNAComposer primarily assembles 3D models from a secondary-structure-driven fragment library and typically returns a limited number of top-ranked low-energy solutions [56]. AlphaFold3, as a diffusion-based learning approach for biomolecular complexes including nucleic acids, similarly prioritizes high-confidence predictions over thermodynamic structure collections [2]. These differences are particularly relevant for our study, since the unbound state includes a flexible single-stranded poly(A)-linker connecting structured domains. Increasing evidence shows that single-stranded nucleic acids can exhibit broad, sequence- and condition-dependent conformational distributions and fast chain dynamics, making accurate modeling of such linkers non-trivial [53]. The broad structural spectrum generated by FARFAR2 can be interpreted as unbound conformations with substantial poly(A)-linker flexibility and conformations in which the tertiary contact is partially formed, i.e. precursor states where the GAAA tetraloop and the KL are in close spatial proximity. The latter correspond to high transfer efficiencies that are not apparent in the experimental distribution, although we note that smFRET may report time-averaged structure collections within the burst time window. The narrower distributions obtained from RNAComposer and AlphaFold3 reflect an unbound state, characterised by an elongated poly(A)-linker, which also figures among the structures predicted by FARFAR2.

Complementing the prediction-derived structure collections, MD simulations provide an independent, physics-based reference for interpreting a FRET distribution of a conformationally flexible RNA. Specifically, they model the dominant degrees of freedom of the poly(A)-linker on a physical timescale of nano- to microseconds. Practically, the MD simulations were initiated from multiple randomized starting orientations of the poly(A)-linker relative to the structured motif of the KL, resulting in an energy-transfer efficiency distribution that overlap with those obtained from FARFAR2 and RNAComposer/AlphaFold3.

To evaluate how well predicted structure collections reproduce experimentally observed FRET signals, we simulated photon bursts using FRETraj [27, 38, 39, 43]. This forward modeling ensures that the simulated FRET values reflect those expected in a real smFRET experiment. In the case of MD trajectories photons were simulated from structures linked on a time dimension. Within a burst, photons were sampled from the same trajectory. Conversely, structures generated by 3D prediction tools are not physically linked in time and thus were treated as if they were rapidly sampled during the photon detection time window. Consequently, the Monte-Carlo simulation models a fast-exchange regime, where the RNA explores the conformational space on short timescales compared to the burst window. The detected FRET signal reflects the corresponding time-averaged efficiencies. The simulated FRET distributions derived from both MD and FARFAR2 converge to similar mean FRET values of 0.65 and 0.77, respectively. This convergence illustrates that both static structural heterogeneity and fast conformational dynamics can yield the same average FRET signal when the motion timescale is fast relative to the burst detection time window. However, neither MD nor FARFAR2 fully reproduce the experimental FRET distribution.

As FARFAR2 generates a broad set of physically plausible conformations, one could hypothesize that a subset of these seemingly incompatible conformations would occur under different ionic conditions than those used in the present experiment. In fact, the higher-FRET conformations may represent precursors states toward a stable tertiary interaction between GAAA and the KL, as observed in the cryo-EM reference structure. This interpretation leads to a potential advantage of sampling-oriented prediction: it can produce conformations that may become relevant under altered conditions (e.g. different cation composition) or along a binding/folding pathway, even if they are not prominently populated in a given solution experiment. Finally, relevant RNA dynamics may also occur on slower timescales (microseconds to milliseconds) than assumed in the fast-exchange photon-burst simulation [57–59]. Longer MD simulations and/or enhanced sampling may be required to access more extended conformations associated with lower transfer efficiencies, as produced by RNAComposer and AlphaFold3.

To reconcile experimental and simulated distributions, we introduced a FRET-guided reweighting strategy. This procedure resamples the structure collections to be compatible with the experimental FRET distribution (Table 1 w/ photon sampling—weighted). A central prerequisite of this approach is that the FRET experiment probes the intended structural state. In the present case, we assume that the measured smFRET trajectory indeed reports on the unbound state, and that the observed FRET dynamics primarily reflect the mobility and spatial exploration of the GAAA tetraloop under the chosen solution conditions. In other words, the reweighting procedure is meaningful only if the FRET observable is sensitive to the conformational transition of interest and if the underlying construct populates the corresponding conformations in solution. Importantly, agreement between predicted and experimental FRET distributions can only be achieved if structural candidates with experimentally compatible $R_{\mathrm{DA}}$ and $E_{\mathrm{DA}}$ values are present in the predicted structure collections. Obviously, reweighting will not generate new conformations, it only redistributes probability mass among conformations that exhibit overlap between the predicted and experimentally observed FRET distributions. Consequently, higher conformational coverage will increase the success rate of reweighting.

After reweighting, all three RNA 3D structure prediction tools and the MD simulation yield FRET distributions with similar means and widths. Among the prediction algorithms, FARFAR2 most closely reproduces the experimental mean FRET efficiency (0.32), though its distribution remains slightly narrower than that observed experimentally, which is a consequence of structural averaging during burst simulation in FRETraj. RNAComposer and AlphaFold3 exclusively covered low-FRET states and thus tended to miss intermediate FRET bins. Additionally, structural entanglements were observed across all prediction methods and may contribute to a small fraction of sterically or topologically implausible conformations (Supplementary Table S4). However, their impact is limited here, because enforcing correct KL formation effectively excludes entanglements involving the linker region.

In summary, our workflow integrates RNA 3D structure prediction with smFRET distance distributions to perform solution-state selection among physically plausible models. We showed that none of the validated structure collections reproduces the low-FRET distribution without reweighting. A key advantage of this approach is that smFRET can efficiently reject implausible candidates and enrich for conformations consistent with the defined experimental conditions. Capturing the highly dynamic nature of RNA [60], thus requires structure prediction approaches that provide sufficiently diverse structure collections. Precise single-molecule measurements impose realistic constraints on this candidate pool, enabling effective re-sampling and selection of concordant conformations. Applying this strategy to larger and more complex RNAs will likely require multiple non-redundant FRET coordinates that report on orthogonal conformational transitions. Overall, this work supports a gradual paradigm shift from picking a single “best” model toward identifying collections of models that are structurally compatible with solution-based experimental observables.

## Supplementary Material

gkag147_Supplemental_File

## Data Availability

The source code of FRETraj is available on GitHub https://github.com/RNA-FRETools/fretraj and Zenodo https://doi.org/10.5281/zenodo.10898652. Documentation can be found at https://rna-fretools.github.io/software/. All 10 000 initial structures predicted by RNAComposer, FARFAR2, and AlphaFold3, the downsampled sets of 1000 structures used for FRET analysis, the six $1 \,{\mu }\mathrm{s}$ MD trajectories initiated from selected seed structures, and all associated ACV files (.pkl) generated via FRETraj are available at Zenodo: https://doi.org/10.5281/zenodo.15971162. Additional source material is available from the corresponding author upon request.

## References

[1] Karplus M, McCammon JA. Molecular dynamics simulations of biomolecules. Nat Struct Biol. 2002;9:646–52. 10.1038/nsb0902-646. 12198485

[2] Abramson J, Adler J, Dunger J et al. Accurate structure prediction of biomolecular interactions with AlphaFold 3. Nature. 2024;630:493–500. 10.1038/s41586-024-07487-w. 38718835 PMC11168924

[3] Nam K, Wolf-Watz M. Protein dynamics: the future is bright and complicated!. Struct Dyn. 2023;10:014301. 10.1063/4.0000179. 36865927 PMC9974214

[4] Hospital A, Goñi JR, Orozco M et al. Molecular dynamics simulations: advances and applications. Adv Appl Bioinform Chem. 2015;8:37–47. 10.2147/AABC.S70333. 26604800 PMC4655909

[5] Statello L, Guo CJ, Chen LL et al. Gene regulation by long noncoding RNAs and its biological functions. Nat Rev Mol Cell Biol. 2021;22:96–118. 10.1038/s41580-020-00315-9. 33353982 PMC7754182

[6] Pucci F, Schug A. Shedding light on the dark matter of the biomolecular structural universe: progress in RNA 3D structure prediction. Methods. 2019;162-163:68–73. 10.1016/j.ymeth.2019.04.012. 31028927

[7] Cruz JA, Blanchet MF, Boniecki M et al. RNA-Puzzles: a CASP-like evaluation of RNA three-dimensional structure prediction. RNA. 2012;18:610–25. 10.1261/rna.031054.111. 22361291 PMC3312550

[8] Miao Z, Adamiak RW, Blanchet MF et al. RNA-Puzzles Round II: assessment of RNA structure prediction programs applied to three large RNA structures. RNA. 2015;21:1066–84. 10.1261/rna.049502.114. 25883046 PMC4436661

[9] Miao Z, Adamiak RW, Antczak M et al. RNA-Puzzles Round III: 3D RNA structure prediction of five riboswitches and one ribozyme. RNA. 2017;23:655–72. 10.1261/rna.060368.116. 28138060 PMC5393176

[10] Miao Z, Adamiak RW, Antczak M et al. RNA-Puzzles Round IV: 3D structure predictions of four ribozymes and two aptamers. RNA. 2020;26:982–95. 10.1261/rna.075341.120. 32371455 PMC7373991

[11] Bu F, Adam YAI, Adamiak R et al. RNA-Puzzles Round V: blind predictions of 23 RNA structures. Nat Methods. 2024;22:399–411. 10.1038/s41592-024-02543-9. 39623050 PMC11810798

[12] Bernard C, Postic G, Ghannay S et al. State-of-the-RNArt: benchmarking current methods for RNA 3D structure prediction. NAR Genom Bioinform. 2024;6:lqae048. 10.1093/nargab/lqae048. 38745991 PMC11091930

[13] Galli S . X ray crystallography: one century of Nobel prizes. J Chem Educ. 2014;91:2009–12. 10.1021/ed500343x.

[14] Ferguson RC, Phillips WD. High-resolution nuclear magnetic resonance spectroscopy. Advances in instrumentation in this field are leading to new applications in chemistry and biology. Science. 1967;157:257–67. 10.1126/science.157.3786.257. 6028395

[15] Subramaniam S, Kleywegt GJ. A paradigm shift in structural biology. Nat Methods. 2022;19:20–3. 10.1038/s41592-021-01361-7. 35017736

[16] Sponer J, Bussi G, Krepl M et al. RNA structural dynamics as captured by molecular simulations: a comprehensive overview. Chem Rev. 2018;118:4177–338. 10.1021/acs.chemrev.7b00427. 29297679 PMC5920944

[17] Svergun DI, Koch MHJ. Small-angle scattering studies of biological macromolecules in solution. Rep Prog Phys. 2003;66:1735–82. 10.1088/0034-4885/66/10/R05.

[18] Tants JN, Schlundt A. Advances, applications, and perspectives in small-angle X-ray scattering of RNA. ChemBioChem. 2023;24:e202300110. 10.1002/cbic.202300110. 37466350

[19] Ponce-Salvatierra A, Astha, Merdas K et al. Computational modeling of RNA 3D structure based on experimental data. Biosci Rep. 2019;39:BSR20180430. 10.1042/BSR20180430. 30670629 PMC6367127

[20] Jeschke G . MMM: a toolbox for integrative structure modeling. Protein Sci. 2018;27:76–85. 10.1002/pro.3269. 28799219 PMC5734387

[21] Förster T . Zwischenmolekulare energiewanderung und fluoreszenz. Ann Phys. 1948;437:55–75. 10.1002/andp.19484370105.

[22] Lerner E, Barth A, Hendrix J et al. FRET-based dynamic structural biology: challenges, perspectives and an appeal for open-science practices. Elife. 2021;10. 10.7554/eLife.60416. PMC8007216 33779550

[23] Hellenkamp B, Schmid S, Doroshenko O et al. Precision and accuracy of single-molecule FRET measurements-a multi-laboratory benchmark study. Nat Methods. 2018;15:669–76. 10.1038/s41592-018-0085-0. 30171252 PMC6121742

[24] Peter MF, Gebhardt C, Mächtel R et al. Cross-validation of distance measurements in proteins by PELDOR/DEER and single-molecule FRET. Nat Commun. 2022;13:4396. 10.1038/s41467-022-31945-6. 35906222 PMC9338047

[25] Dimura M, Peulen TO, Hanke CA et al. Quantitative FRET studies and integrative modeling unravel the structure and dynamics of biomolecular systems. Curr Opin Struct Biol. 2016;40:163–85. 10.1016/j.sbi.2016.11.012. 27939973

[26] Vallat B, Webb B, Westbrook JD et al. Development of a prototype system for archiving integrative/hybrid structure models of biological macromolecules. Structure. 2018;26:894–904. 10.1016/j.str.2018.03.011. 29657133 PMC5990459

[27] Steffen FD, Cunha RA, Sigel RKO et al. FRET-guided modeling of nucleic acids. Nucleic Acids Res. 2024;52:e59. 10.1093/nar/gkae496. 38869063 PMC11260485

[28] Woodson SA . RNA folding pathways and the self-assembly of ribosomes. Acc Chem Res. 2011;44:1312–19. 10.1021/ar2000474. 21714483 PMC4361232

[29] Leontis NB, Westhof E. Geometric nomenclature and classification of RNA base pairs. RNA. 2001;7:499–512. 10.1017/s1355838201002515. 11345429 PMC1370104

[30] Freisinger E, Sigel RKO. From nucleotides to ribozymes—a comparison of their metal ion binding properties. Coord Chem Rev. 2007;251:1834–51. 10.1016/j.ccr.2007.03.008.

[31] Woodson SA . Taming free energy landscapes with RNA chaperones. RNA Biol. 2010;7:677–86. 10.4161/rna.7.6.13615. 21045544 PMC3073327

[32] Gerhardy S, Oborská-Oplová M, Gillet L et al. Puf6 primes 60S pre-ribosome nuclear export at low temperature. Nat Commun. 2021;12:4696. 10.1038/s41467-021-24964-2. 34349113 PMC8338941

[33] Dimura M, Peulen TO, Sanabria H et al. Automated and optimally FRET-assisted structural modeling. Nat Commun. 2020;11:5394. 10.1038/s41467-020-19023-1. 33106483 PMC7589535

[34] Hanke CA, Westbrook JD, Webb BM et al. Making fluorescence-based integrative structures and associated kinetic information accessible. Nat Methods. 2024;21:1970–72. 10.1038/s41592-024-02428-x. 39349602 PMC11939118

[35] Popenda M, Szachniuk M, Antczak M et al. Automated 3D structure composition for large RNAs. Nucleic Acids Res. 2012;40:e112. 10.1093/nar/gks339. 22539264 PMC3413140

[36] Sarzynska J, Popenda M, Antczak M et al. RNA tertiary structure prediction using RNAComposer in CASP15. Proteins. 2023;91:1790–9. 10.1002/prot.26578. 37615316

[37] Watkins AM, Rangan R, Das R. FARFAR2: improved *de novo* Rosetta prediction of complex global RNA folds. Structure. 2020;28:963–76. 10.1016/j.str.2020.05.011. 32531203 PMC7415647

[38] Steffen FD, Sigel RKO, Börner R. An atomistic view on carbocyanine photophysics in the realm of RNA. Phys Chem Chem Phys. 2016;18:29045–55. 10.1039/C6CP04277E. 27783069

[39] Steffen FD, Sigel RKO, Börner R. FRETraj: integrating single-molecule spectroscopy with molecular dynamics. Bioinformatics. 2021;37:3953–5. 10.1093/bioinformatics/btab615. 34478493 PMC10186158

[40] Bottaro S, Di Palma F, Bussi G. The role of nucleobase interactions in RNA structure and dynamics. Nucleic Acids Res. 2014;42:13306–14. 10.1093/nar/gku972. 25355509 PMC4245972

[41] Bottaro S, Bussi G, Pinamonti G et al. Barnaba: software for analysis of nucleic acid structures and trajectories. RNA. 2019;25:219–231. 10.1261/rna.067678.118. 30420522 PMC6348988

[42] Hoefling M, Lima N, Haenni D et al. Structural heterogeneity and quantitative FRET efficiency distributions of polyprolines through a hybrid atomistic simulation and Monte Carlo approach. PLoS One. 2011;6:e19791. 10.1371/journal.pone.0019791. 21629703 PMC3101224

[43] Hoefling M, Grubmueller H. *In silico* FRET from simulated dye dynamics. Comput Phys Commun. 2013;184:841–52. 10.1016/j.cpc.2012.10.018.

[44] Kullback S, Leibler RA. On information and sufficiency. Ann Math Stat. 1951;22:79–86. 10.1214/aoms/1177729694.

[45] Erichson F, Steffen FD, Börner R. FAMP: a software framework for FRET-based integrative modeling of RNA. In: Arluison V, Wien F (eds.), *RNA Spectroscopy: Methods and Protocols. Methods in Molecular Biology*, Vol. 3004. New York, NY: Humana, 2026. 10.1007/978-1-0716-5084-4_2. 41478898

[46] Leontis NB, Zirbel CL. Nonredundant 3D structure datasets for RNA knowledge extraction and benchmarking. In: Leontis N, Westhof E (eds.),*RNA 3D Structure Analysis and Prediction. Nucleic Acids and Molecular Biology*, Vol. 27. Berlin, Heidelberg: Springer, 2012, 281–98. 10.1007/978-3-642-25740-7_13.

[47] Erichson F, Steffen FD, Börner R, FRET-assisted structural model of the GAAA RNA tetraloop receptor. In: *26. Interdisziplinäre Wissenschaftliche Konferenz Mittweida*, Vol. 002. Mittweida: Hochschuldruckerei Hochschule Mittweida, 2021, 230–3. 10.48446/opus-12283.

[48] Parks JW, Kappel K, Das R et al. Single-molecule FRET-Rosetta reveals RNA structural rearrangements during human telomerase catalysis. RNA. 2017;23:175–88. 10.1261/rna.058743.116. 28096444 PMC5238793

[49] Li J, Walter NG, Chen SJ. smFRET-assisted RNA structure prediction. Commun Inf Syst. 2024;24:163–79. 10.4310/cis.241021213225. 39524454 PMC11545564

[50] Hodak JH, Fiore JL, Nesbitt DJ et al. Docking kinetics and equilibrium of a GAAA tetraloop-receptor motif probed by single-molecule FRET. Proc Natl Acad Sci USA. 2005;102:10505–10. 10.1073/pnas.0408645102. 16024731 PMC1180751

[51] Sindbert S, Kalinin S, Nguyen H et al. Accurate distance determination of nucleic acids via Förster resonance energy transfer: Implications of dye linker length and rigidity. J Am Chem Soc. 2011;133:2463–80. 10.1021/ja105725e. 21291253

[52] Barth A, Opanasyuk O, Peulen TO et al. Unraveling multi-state molecular dynamics in single-molecule FRET experiments. I. Theory of FRET-lines. J Chem Phys. 2022;156:141501. 10.1063/5.0089134. 35428384 PMC9014241

[53] Nüesch MF, Pietrek L, Holmstrom ED et al. Nanosecond chain dynamics of single-stranded nucleic acids. Nat Commun. 2024;15:6010. 10.1038/s41467-024-50092-8. 39019880 PMC11255343

[54] Downey CD, Fiore JL, Stoddard CD et al. Metal ion dependence, thermodynamics, and kinetics for intramolecular docking of a GAAA tetraloop and receptor connected by a flexible linker. Biochemistry. 2006;45:3664–73. 10.1021/bi0520941. 16533049 PMC2735227

[55] Willemsen AM, Van Os GA. Interaction of magnesium ions with poly A and poly U. Biopolymers. 1971;10:945–60. 10.1002/bip.360100602. 5092618

[56] Biesiada M, Pachulska-Wieczorek K, Adamiak RW et al. RNAComposer and RNA 3D structure prediction for nanotechnology. Methods. 2016;103:120–27. 10.1016/j.ymeth.2016.03.010. 27016145

[57] Nir E, Michalet X, Hamadani KM et al. Shot-noise limited single-molecule FRET histograms: comparison between theory and experiments. J Phys Chem B. 2006;110:22103–24. 10.1021/jp063483n. 17078646 PMC3085016

[58] Kalinin S, Sisamakis E, Magennis SW et al. On the origin of broadening of single-molecule FRET efficiency distributions beyond shot noise limits. J Phys Chem B. 2010;114:6197–206. 10.1021/jp100025v. 20397670

[59] Schuler B, Hofmann H. Single-molecule spectroscopy of protein folding dynamics—expanding scope and timescales. Curr Opin Struct Biol. 2013;23:36–47. 10.1016/j.sbi.2012.10.008. 23312353

[60] Ganser LR, Kelly ML, Herschlag D et al. The roles of structural dynamics in the cellular functions of RNAs. Nat Rev Mol Cell Biol. 2019;20:474–89. 10.1038/s41580-019-0136-0. 31182864 PMC7656661

