# Supplementary material for: FRET-guided selection of RNA 3D structures
Source: Nucleic Acids Res. 2026 Feb 25;54(5):gkag147. doi: 10.1093/nar/gkag147 (PMC12956335; doi:10.1093/nar/gkag147)
Supplement: gkag147_Supplemental_File [file gkag147_supplemental_file.pdf]

Supplementary Information

FRET-guided selection of RNA 3D structures

Mirko Weber[1], Felix Erichson[1], Maciej Antczak[2,3], Vanessa Schumann[1], Josephine Meitzner[1], Tomasz Zok[3], Fabio D. Steffen[4], Marta Szachniuk[2,3], and Richard Börner[1,*]

[1]Laserinstitut Hochschule Mittweida, University of Applied Sciences Mittweida, Technikumplatz 17, 09648, Mittweida, Germany
[2]Institute of Bioorganic Chemistry, Polish Academy of Sciences, Noskowskiego 12/14, 61-704, Poznan, Poland
[3]Institute of Computing Science, Poznan University of Technology, Piotrowo 2, 60-965, Poznan, Poland
[4]Department of Oncology, University of Zurich, University Children's Hospital, 8008, Zurich, Switzerland
[*]Corresponding author: richard.boerner@hs-mittweida.de - Tel: +49 3727 58 1009

January 13, 2026

Supplementary Methods

smFRET

To ensure kissing loop formation of the KL-TL$_{\mathrm{GAAA}}$ rRNA model construct, the fluorescently labeled RNA (purchased from IBA) was imaged free in solution in the presence of 116 mM KCl, buffered in 50 mM Tris-HCl at pH 7.5 and room temperature (1). The single-molecule measurement was performed on a home-built confocal microscope equipped with a $N_{\mathrm{A}} = 1.2$ water-immersion objective (Nikon), a Timeharp 260 TSCPC card (Picoquant), a 532 nm cw (40 μW) and a 638 nm pulsed laser source (6 μW). The latter uses a 10 MHz repetition rate for pulsed overleaved excitation (POE) allowing molecular sorting.(2) Photons were spectrally separated donor 582/64 and acceptor channel 690/70 band pass filters and detected onto two avalanche photodiodes (Perkin Elmer). The data was analysed using a sliding time window with a two-color, all-photon burst search, which identified bursts with a total photon count of at least 40 ($N_{\mathrm{D}} + N_{\mathrm{A}}$). Double-labeled molecules were selected by applying an intensity threshold of red photons after acceptor excitation ($N_{\mathrm{A,A}} > 20$) to remove the donor-only population and a stoichiometry limit $S > 0.2$ to eliminate any acceptor-only species (3). FRET histograms were corrected according to standard protocols (4, 5). smFRET correction factors are summarized in Supplementary Table S1. The gamma factor of the home-built fluorescence microscope was determined according to McCann et al. (6). Therein, we calibrated the detection efficiency of the donor and acceptor channel with a FluoroMax spectral photometer from Horiba. We determined the quantum yield of the FRET pair for the fluorescent labeled RNA with an ID5 spectral photometer from Molecular Devices. As reference dyes we used rhodamine 6G and Atto647N dissolved in ethanol at μM concentrations. The Förster radius of the FRET pair Cy3/Cy5 $R_0 = 6.17$ nm was determined with the donor quantum yield and the spectral overlap integral of the FRET dye pair (data not shown).

Structure collection prediction with RNAComposer

To obtain 10,000 unique *in silico* models of the KL-TL$_{\text{GAAA}}$ construct (1), we used the RNAComposer web server in batch mode (available to logged-in users) at https://rnacomposer.cs.put.poznan.pl/ (7). We submitted twelve identical batches ($\{batch\_no\}\ i = 1 \ldots 12$), each consisting of $j = 1 \ldots 10$ identical tasks. Each task was defined by a single RNA sequence and ten identical secondary structures $k = 1 \ldots 10$. An example batch is shown below:

```
>KLTL{batch_no}i
#j = 1
UGAAGAAAUUCAAAAAAAAAGCUCGGAAUUUGAGCAAAAAAAAAAAACGGUGGUAAAUUCCAUCG
#k = 1
((((....))))........((((.[[[[[[))))............(((((..]]]]]])))))
...
#k = 10
((((....))))........((((.[[[[[[))))............(((((..]]]]]])))))

...

#j = 10
UGAAGAAAUUCAAAAAAAAAGCUCGGAAUUUGAGCAAAAAAAAAAAACGGUGGUAAAUUCCAUCG
#k = 1
((((....))))........((((.[[[[[[))))............(((((..]]]]]])))))
...
#k = 10
((((....))))........((((.[[[[[[))))............(((((..]]]]]])))))
```

The input was submitted 1,200 times across all batches (10 inputs per task × 10 tasks per batch × 12 batches). For each such input, RNAComposer generated a family of ten 3D structure collections. The first model in each family was constructed using the highest-scoring structural elements, selected deterministically according to RNAComposer's internal scoring function. The remaining nine models were generated by randomly assembling well-scoring alternative elements (where sequence similarity remains highest, but we allow, e.g., lower experimental resolution), making the procedure non-deterministic. Consequently, for two identical inputs, the first predicted model would be the same, while the remaining nine would, with high probability, differ. As a result of processing 1,200 identical inputs, we obtained 12,000 RNA 3D models, of which 1,200 were identical (the first models from each family) and 10,800 were structurally diverse. The corresponding ZIP files containing all 12,000 models were downloaded from the RNAComposer web server workspace and stored in the '*models*' folder. Filtering out identical structures was performed later in the processing pipeline. From the downloaded archives, we extracted only the PDB files and then removed the ZIP files using the following command:

```
find . -name "*.zip" -exec unzip -d . -j {} "*/*.pdb" \;; rm *.zip
```

We then split multi-model PDB files into individual model files using the following AWK script:

```
BEGIN { filename = ARGV[1]; gsub(/" "/, "-",filename);
split(filename, elems, ".");}
$1 == "MODEL" {file = elems[1] "#" $2 ".pdb";content="";}
```

```
$1 == "ATOM" || $1 == "HETATM" || $1 == "TER" {content = content $0 "\n";}
$1 == "ENDMDL"  {printf("%s",content) > file;}
```

The script was saved as `split` and applied within the '*models*' folder using the command:

```
for i in *.pdb; do awk -f split "$i"; done;
find . -type f ! -name "*#*.pdb" -exec rm {} \;
```

To remove duplicate 3D structures from the prediction set, we used the `fdupes` tool. First, we created a copy of the full set of models in a new folder:

```
cp models models-without-duplicates
```

Then, we applied `fdupes` to identify and remove redundant structures:

```
fdupes -rdN models-without-duplicates > ./fdupes.log
```

This procedure reduced the dataset to 10,801 unique 3D models, which were stored in the '*models-without-duplicates*' folder. From this set, we randomly selected 10,000 structures for further analysis and copied them to the final folder named '*rnacomposer-kl-tlgaaa*' using the following commands:

```
mkdir rnacomposer-kl-tlgaaa;
find models-without-duplicates -mindepth 1 -maxdepth 1 -name '*.pdb' -print0 |
shuf -n 10000 -z | xargs -r0 cp -t rnacomposer-kl-tlgaaa;
```

These 10,000 unique RNA 3D models predicted by RNAComposer were used in the analyses described in this study.

Structure collection prediction with FARFAR2

For 3D RNA structure prediction of the KL-TL_{GAAA} model construct, we used FARFAR2 (8) from the Rosetta Commons toolbox (https://rosettacommons.org/), incorporating sequence and secondary structure constraints to generate 10,000 individual structures. The structure generation process was adapted from the Supplementary Information of Steffen et al. (9). The following command was executed using `rna_denovo`:

```
rna_denovo.linuxgccrelease -nstruct 10000 -fasta kltlgaaa_sequence.fasta
-secstruct_file kltlgaaa_secstruct.txt -silent kltlgaaa.out
-minimize_rna true -cycles 20000
```

The contents of `kltlgaaa_sequence.fasta` and `kltlgaaa_secstruct.txt` files were as follows:

```
> KL-TLGAAA construct
ugaagaaauucaaaaaaaaagcucggaauuugagcaaaaaaaaaaaacggugguaaauuccaucg

((((....))))........((((.[[[[[[))))............(((((..]]]]]])))))
```

The 10,000 structures were subsequently extracted using `rna_denovo` and merged into a single PDB file with the following commands:

```
grep "^SCORE:" kltlgaaa.out | grep -v description | awk '{print $NF ": " $2}' |
tee all_models.txt

extract_pdbs.linuxgccrelease -in:file:silent kltlgaaa.out -tags
`cat all_models.txt | cut -f1 -d':'`

for pdb in `cat all_models.txt | cut -f1 -d':'`; do
  echo MODEL $i >> kltlgaaa.pdb
  echo TITLE "$pdb" >> kltlgaaa.pdb
  cat "$pdb".pdb >> kltlgaaa.pdb
  echo -e "ENDMDL\n\n" >> kltlgaaa.pdb
  i=$(($i+1))
done
```

Structure collection prediction with AlphaFold3

For structure prediction using AlphaFold3 (10) (https://github.com/google-deepmind/alphafold3), 10,000 structures were computed locally using Docker, utilizing the model weights distributed by AlphaFold for non-commercial use (see installation instructions: https://github.com/google-deepmind/alphafold3/blob/main/docs/installation.md). The following input JSON file was used to generate 10,000 predicted structures with AlphaFold3, where `modelSeeds` was defined as a list ranging from 1 to 10,000.

```
{
  "name": "KLTL_GAAA",
  "modelSeeds": [1, ..., 10000],
  "sequences": [
    {
      "rna": {
        "id": "A",
        "sequence": "
            UGAAGAAAUUCAAAAAAAAAGCUCGGAAUUUGAGCAAAAAAAAAAAACGGUGGUAAAUUCCAUCG"
      }
    }
  ],
  "dialect": "alphafold3",
  "version": 2
}
```

MD simulations

Molecular dynamics (MD) simulations were performed using GROMACS 2024.2 (11) with the AMBER force field (12), incorporating parmbsc0 (13) and χOL3 (14, 15) corrections for RNA. The RNA was solvated in a dodecahedral water box with TIP3P or TIP4P (16), charge-neutralized with KCl, and equilibrated at 298 K and 1 bar. In total, six MD simulations were conducted using TIP4P water, each lasting 1 μs. All MD simulations were analyzed using Barnaba to assess the preservation of WC base-pairings in the kissing loop of the model construct. The seed structures for the six MD simulations were derived from the cryo-EM reference structure (PDB ID: 3JCT (17)), truncated to the tertiary contact core region comprising helices H22, H68, and H88. Nucleotides within the KL region that deviated from the target sequence were mutated using PyMOL (18) (https://www.pymol.org/). A poly(A)-linker connecting H22 and H88 was constructed in PyMOL

Builder and manually integrated into the model to extend beyond the KL receptor site. The tetraloop from the reference structure was manually placed at six distinct positions, yielding six structures with identical KL domains. The poly(A)-linker between H88 and H68 was also generated using the PyMOL Builder and subsequently adjusted to connect each tetraloop configuration to the KL domain.

FRETraj Dye parameters

The following FRETraj (19) dye parameters were used to compute the mACVs for all structures, serving as the basis for calculating E_{DA} values and generating the corresponding FRET efficiency histograms. Atom IDs had to be adjusted for each 3D prediction tool because the PDB files use different atom numbering approaches. The CV fraction was determined according to (9, 20). The fluorescence lifetime and dynamic anisotropy measurements for both dyes of the labeled RNA species are shown in Supplementary Figure S3; fitting results are presented in Supplementary Tables S2 and S3.

```
{"Position":
   {"Cy3-65-O3'":
       {"attach_id": <ATOM-ID in PDB>,
            "mol_selection": "all",
            "linker_length": 20,
            "linker_width": 3.5,
            "dye_radius1": 8,
            "dye_radius2": 3,
            "dye_radius3": 1.5,
            "cv_fraction": 0.68,
            "cv_thickness": 3,
            "use_LabelLib": false,
            "grid_spacing": 1.0,
            "simulation_type": "AV3",
            "state": 1,
            "frame_mdtraj": 0,
            "contour_level_AV": 0,
            "contour_level_CV": 0.7,
            "b_factor": 100,
            "gaussian_resolution": 2,
            "grid_buffer": 2.0,
        },
   "Cy5-10-C5":
       {"attach_id": <ATOM-ID in PDB>,
        "mol_selection": "all",
        "linker_length": 20,
        "linker_width": 3.5,
        "dye_radius1": 9.5,
        "dye_radius2": 3,
        "dye_radius3": 1.5,
        "cv_fraction": 0.33,
        "cv_thickness": 3,
        "use_LabelLib": false,
        "grid_spacing": 1.0,
        "simulation_type": "AV3",
        "state": 1,
        "frame_mdtraj": 0,
        "contour_level_AV": 0,
        "contour_level_CV": 0.7,
        "b_factor": 100,
        "gaussian_resolution": 2,
        "grid_buffer": 2.0,
        },
"Distance": {"Cy3-Cy5":
               {"R0": 61.7,
                "n_dist": 1000000}
            }
}
```

FRETraj photon sampling parameters

The following parameters were used for photon sampling with FRETraj across all structure collections to generate both unweighted and weighted FRET distributions, using the corresponding Rkappa files.

```
{
    "dyes": {
        "tauD": 1.4,
        "tauA": 1.12,
        "QD": 0.46,
        "QA": 0.35,
        "etaA": 1,
        "etaD": 0.37,
        "dipole_angle_abs_em": 0
    },
    "sampling": {
        "nbursts": 20000,
        "skipframesatstart": 0,
        "skipframesatend": 0,
        "multiprocessing": true
    },
    "fret": {
        "R0": 61.7,
        "kappasquare": 0.6666,
        "gamma": true,
        "quenching_radius": 1
    },
    "species": {
        "name": ["all"],
        "unix_pattern_rkappa": ["<unweighted> or <weighted> r_kappa file"],
        "unix_pattern_don_coords": [],
        "unix_pattern_acc_coords": [],
        "probability": [1],
        "n_trajectory_splits": null
    },
    "bursts": {
        "lower_limit": null,
        "upper_limit": null,
        "lambda": null,
        "QY_correction": false,
        "averaging": "trajectory",
        "burst_size_file": "<experimental burst sizes>"
    }
}
```

Supplementary Figures

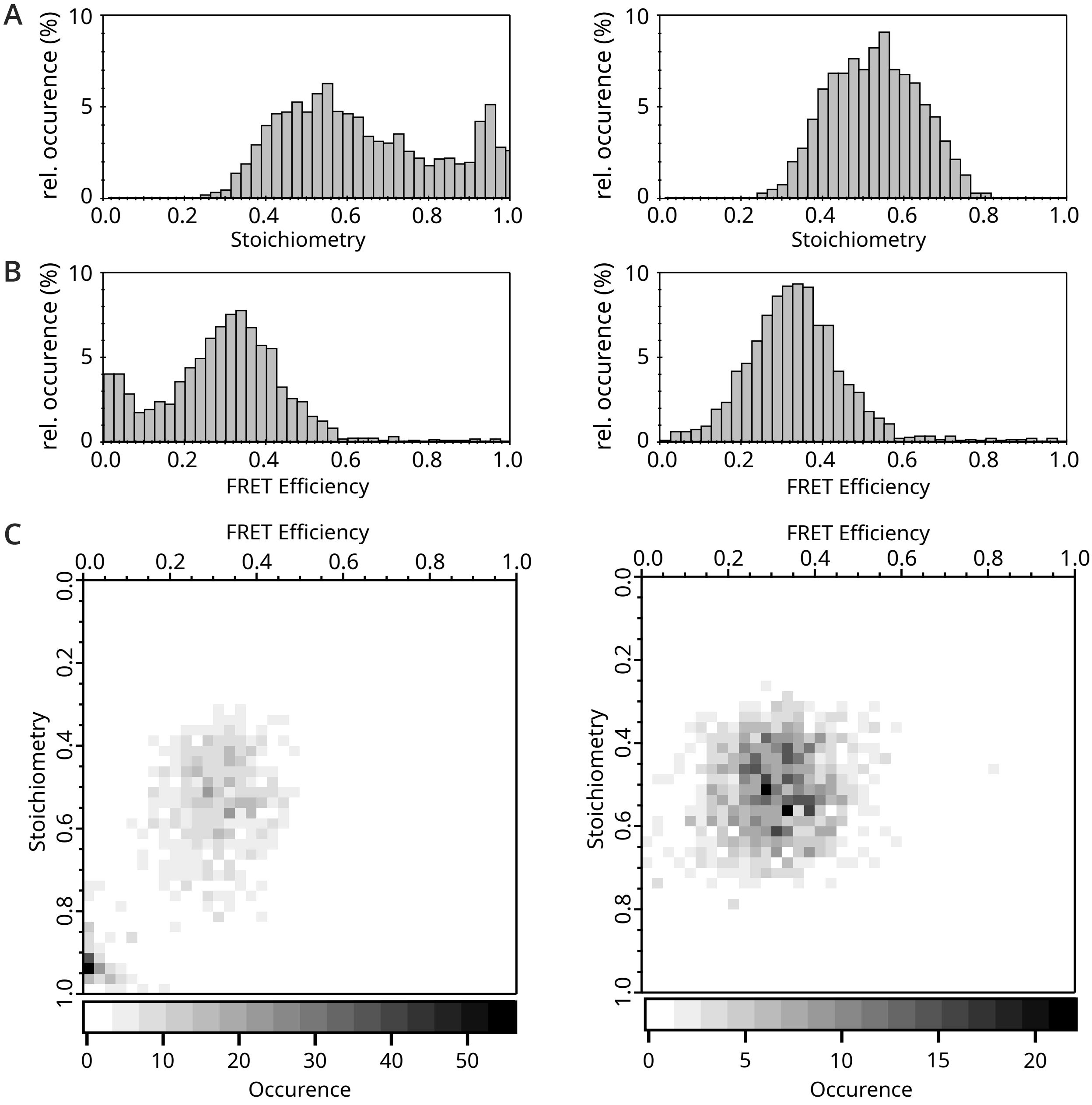

Fig. S1: KL-TL$_{\text{GAAA}}$ probing the GAAA binding in the presence of 116mM K(I) and in 20 mM HEPES buffer, pH 7.5 at RT according to Gerhardy et al. (1). **(A)** Stoichiometry, **(B)** FRET and **(C)** Stoichiometry-FRET histograms (left) without and (right) with molecular sorting based on an acceptor burst intensity threshold of 20 counts per burst. Crosstalk, i.e., bleed-through and direct excitation, background, and gamma factor, has been corrected according to standard protocols. The acceptor-only distribution is negligible.

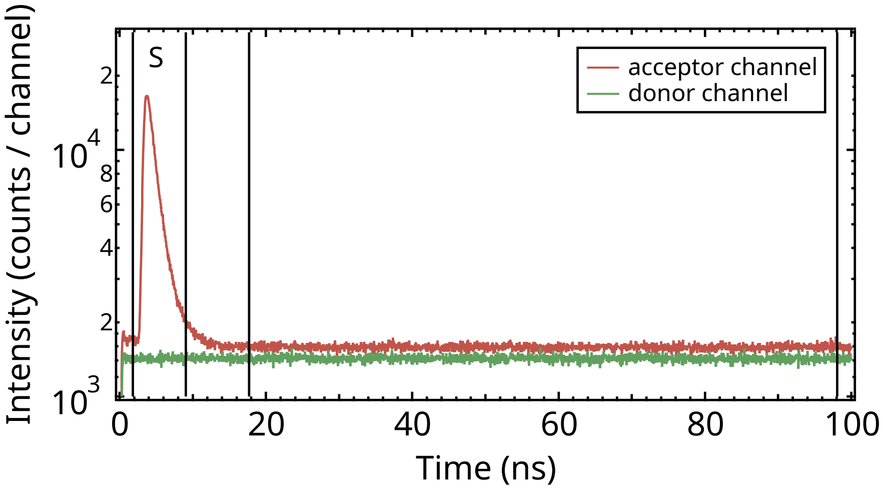

Fig. S2: Pulse overlayed excitation (POE) scheme. The continuous-wave donor excitation overlays an acceptor excitation pulse (10 MHz) to probe the presence of the acceptor dye, thereby enabling molecular sorting. The (micro) time range for stoichiometry and FRET calculations is highlighted.

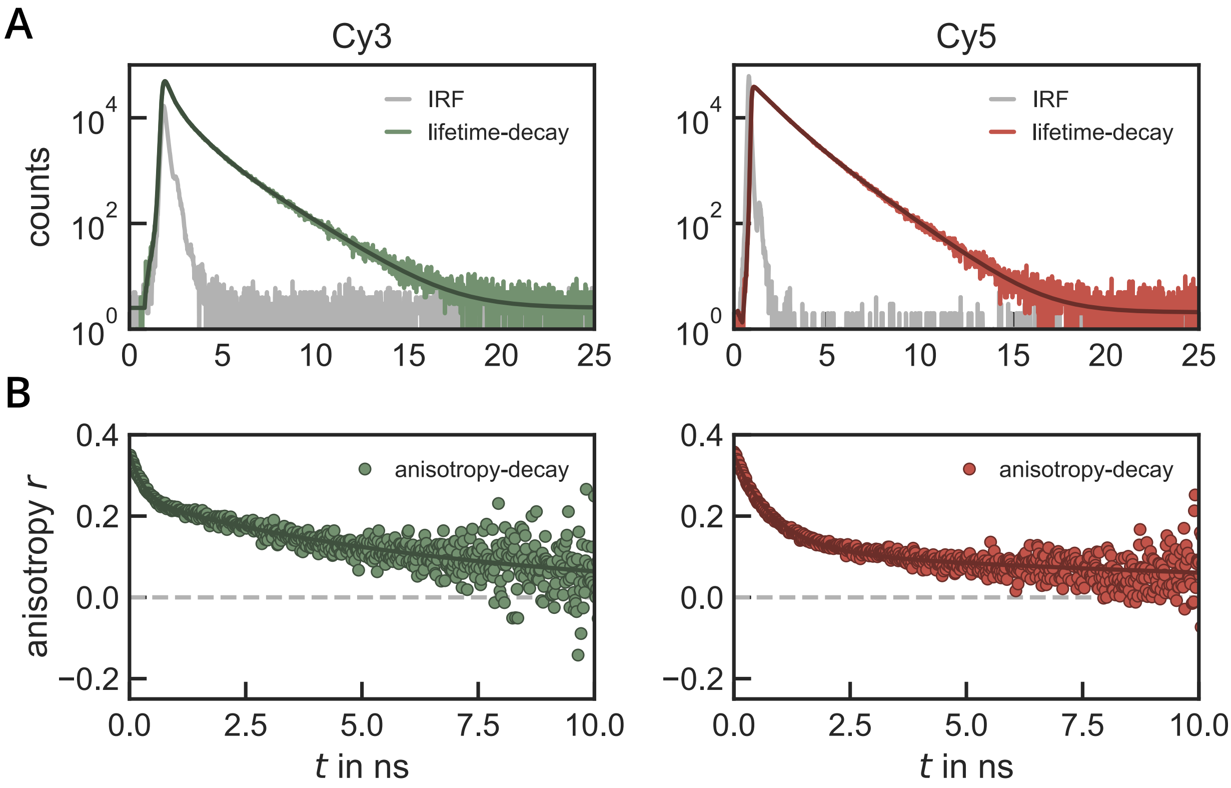

Fig. S3: Fluorescence lifetimes and time-resolved anisotropy decays of Cy3 and Cy5 bound to the KL-TL$_{\mathrm{GAAA}}$ construct in the unbound state (KL formed, GAAA unbound). **(A)** Fluorescence lifetime decays of Cy3 and Cy5, including the instrument response function (IRF), with fits obtained using EasyTau2. **(B)** Anisotropy decays of Cy3 and Cy5 fitted with the local–global rotation-in-a-cone model (20), yielding χ values of 0.68 (Cy3) and 0.33 (Cy5).

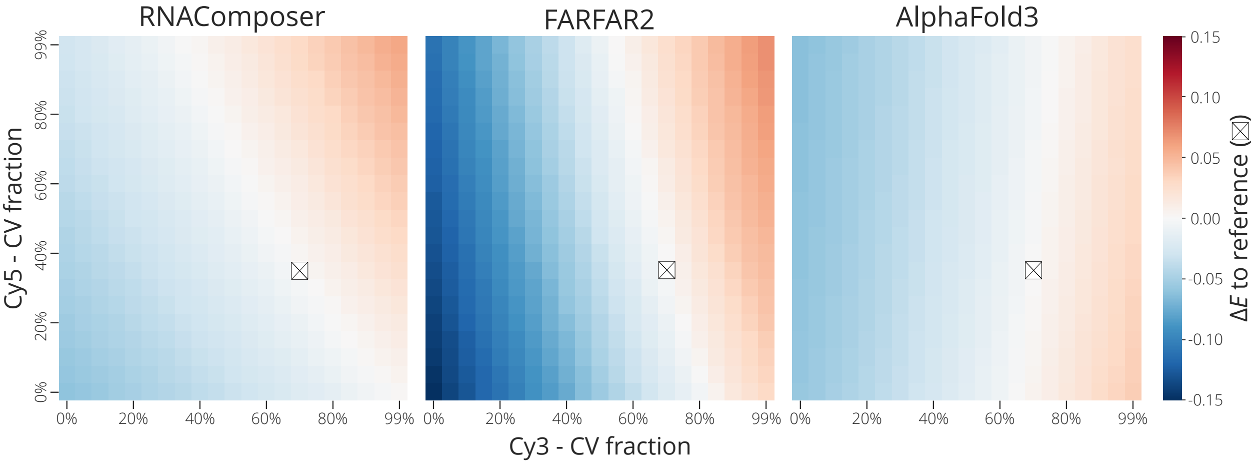

Fig. S4: Effect of the contact volume (CV) to accessible volume (AV) ratio on predicted mean FRET for structures generated by the three RNA 3D prediction tools (also see (9) Figure 2 E-I). The reference is the χ value obtained from fluorescence anisotropy experiments for both dyes. For each AV/CV fraction combination (0.05 steps), the mean FRET was calculated and plotted against the reference, highlighting the importance of dye-specific free/stacked fractions.

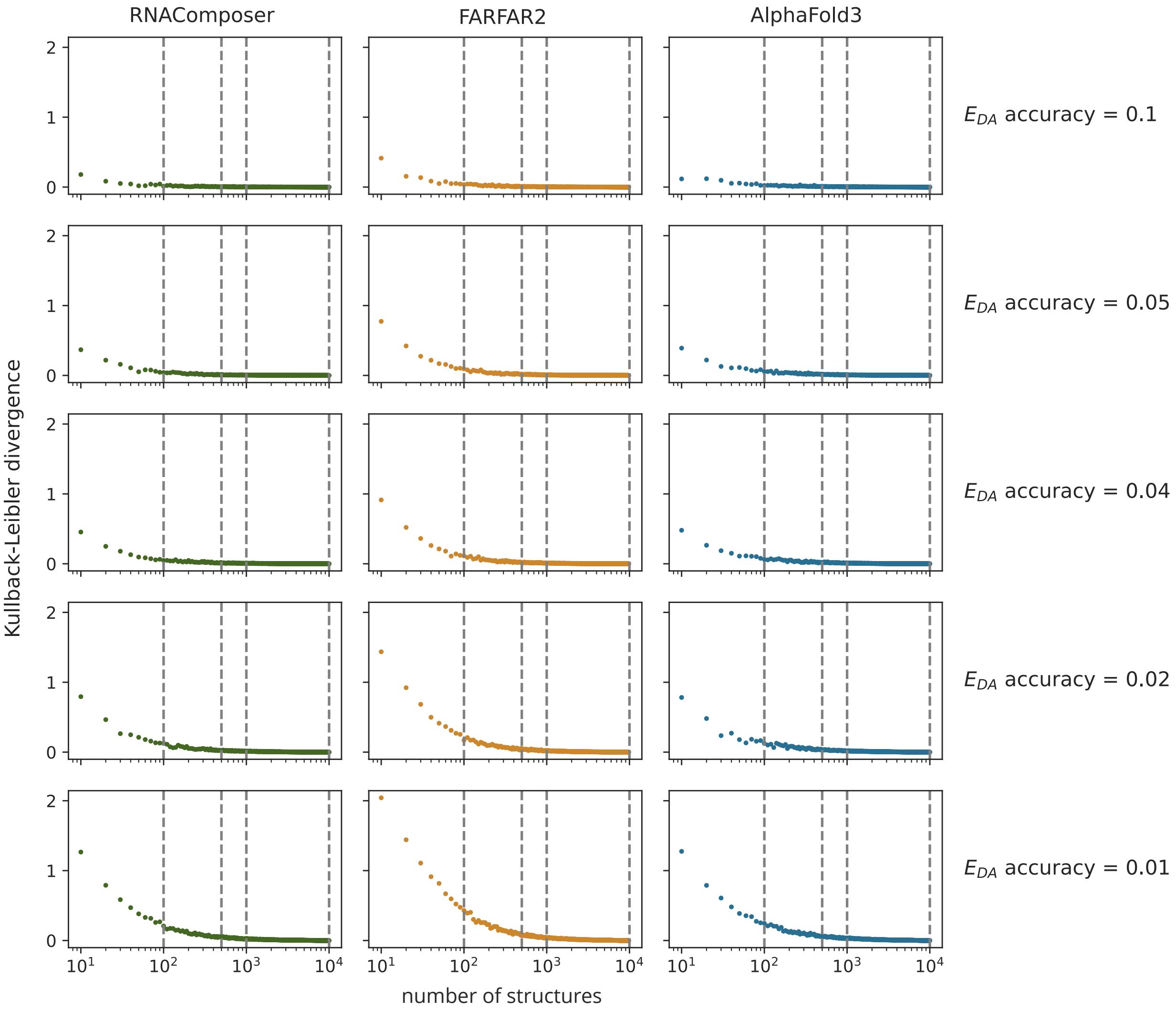

Fig. S5: Dependence of Kullback-Leibler divergence (KLD) on the number of structures in the structure collections generated by RNAComposer, FARFAR2, and AlphaFold3. The E_{DA} accuracy reflects the quality of FRET-based predictions and is affected by the bin size used for computing KLD. At an E_{DA} accuracy of 0.1, as few as 100 structures suffice to represent a full structure collection of 10,000 structures across all tools, whereas an accuracy of 0.01 requires around 1,000 structures.

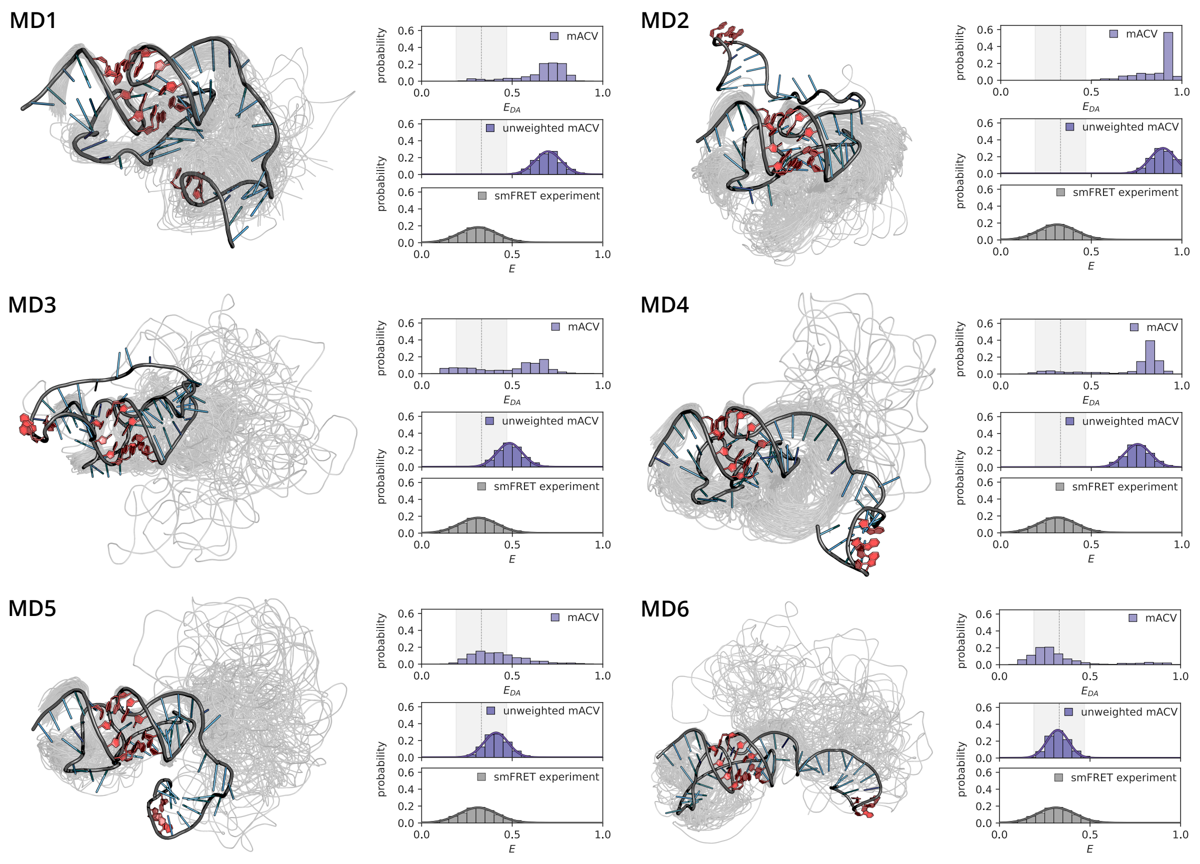

Fig. S6: Seed structures used as starting points for the MD simulations. For each simulation, the input structure and ribbon conformations sampled every 10 ns are shown. Additionally, the corresponding E_{DA} distributions and unweighted FRET distributions obtained after photon sampling are plotted and compared with the experimental smFRET histogram.

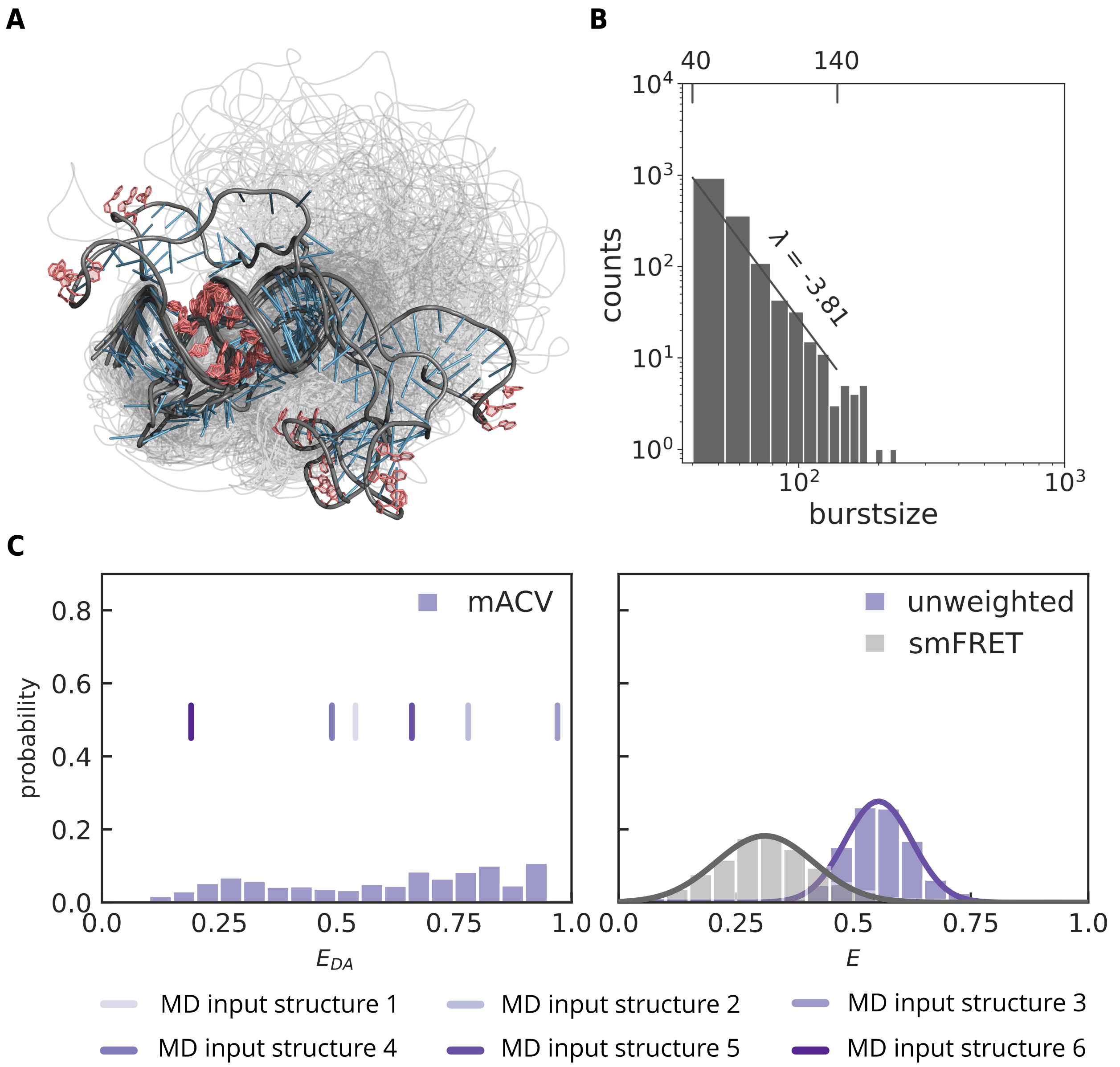

Fig. S7: Six seed structures treated as one structure collection, with corresponding simulated FRET distributions. **(A)** shows the seed structures as well as the states, which were randomly chosen from all six simulations. **(B)** displays the experimental burst size distribution used for the calculation of the *in silico* burst simulation with FRETraj. **(C)** shows the E_{DA} histogram for the combined MD simulation states, as well as the six input E_{DA} values of the seed structures on the left side. The right side shows the E histogram (with photon sampling) plotted against the experimental smFRET histogram.

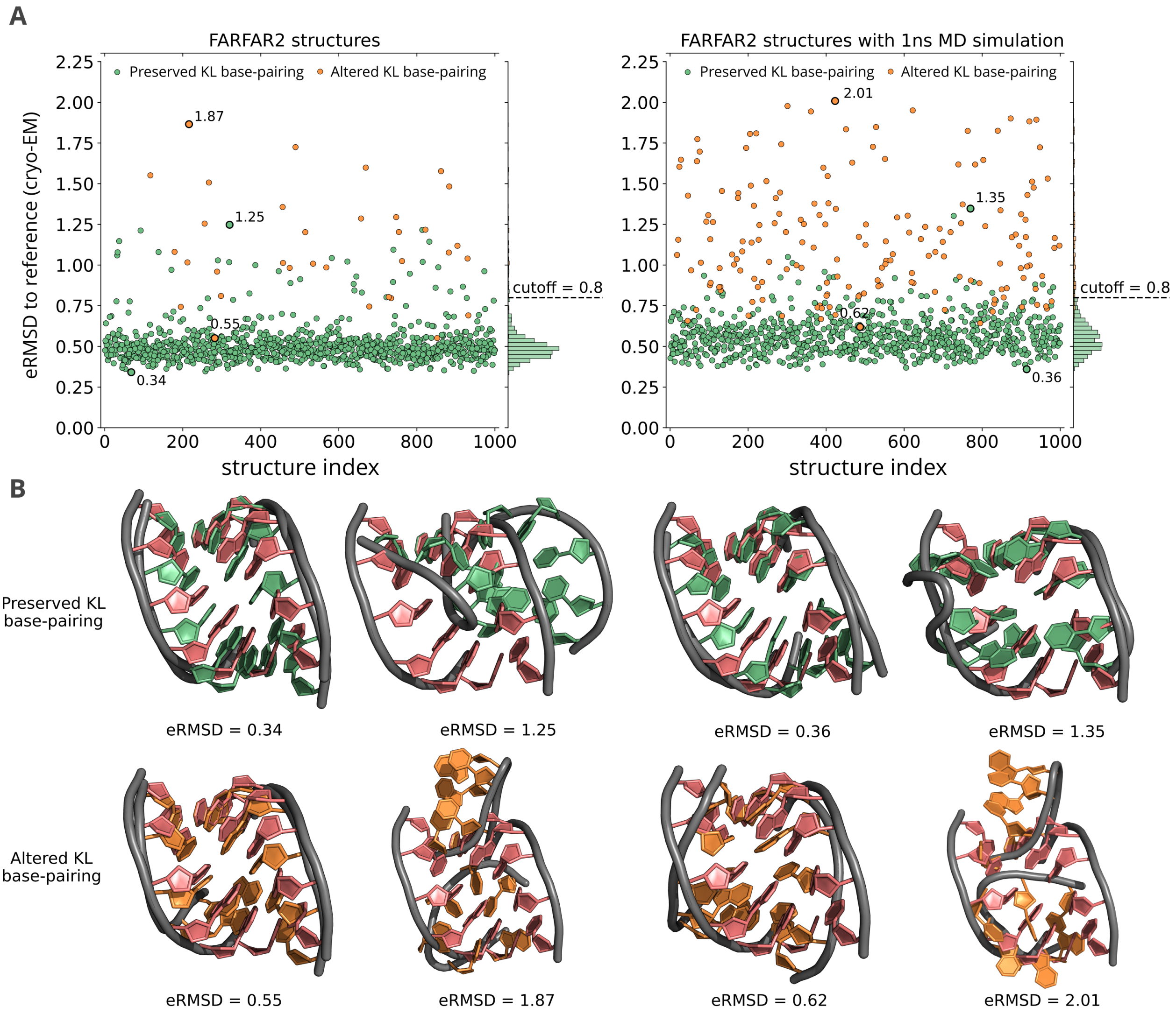

Fig. S8: Correct kissing loop formation and eRMSD values for the FARFAR2 structure collection before and after 1ns MD simulation. **(A)** eRMSD of all structures from the FARFAR2 collection before (left) and after (right) 1 ns MD simulation, relative to the reference cryo-EM structure, focusing on the kissing loop region. **(B)** Representative structures classified as correctly forming the kissing loop (with the lowest and highest eRMSD), and incorrectly forming the kissing loop (also with the lowest and highest eRMSD), both before and after MD simulation.

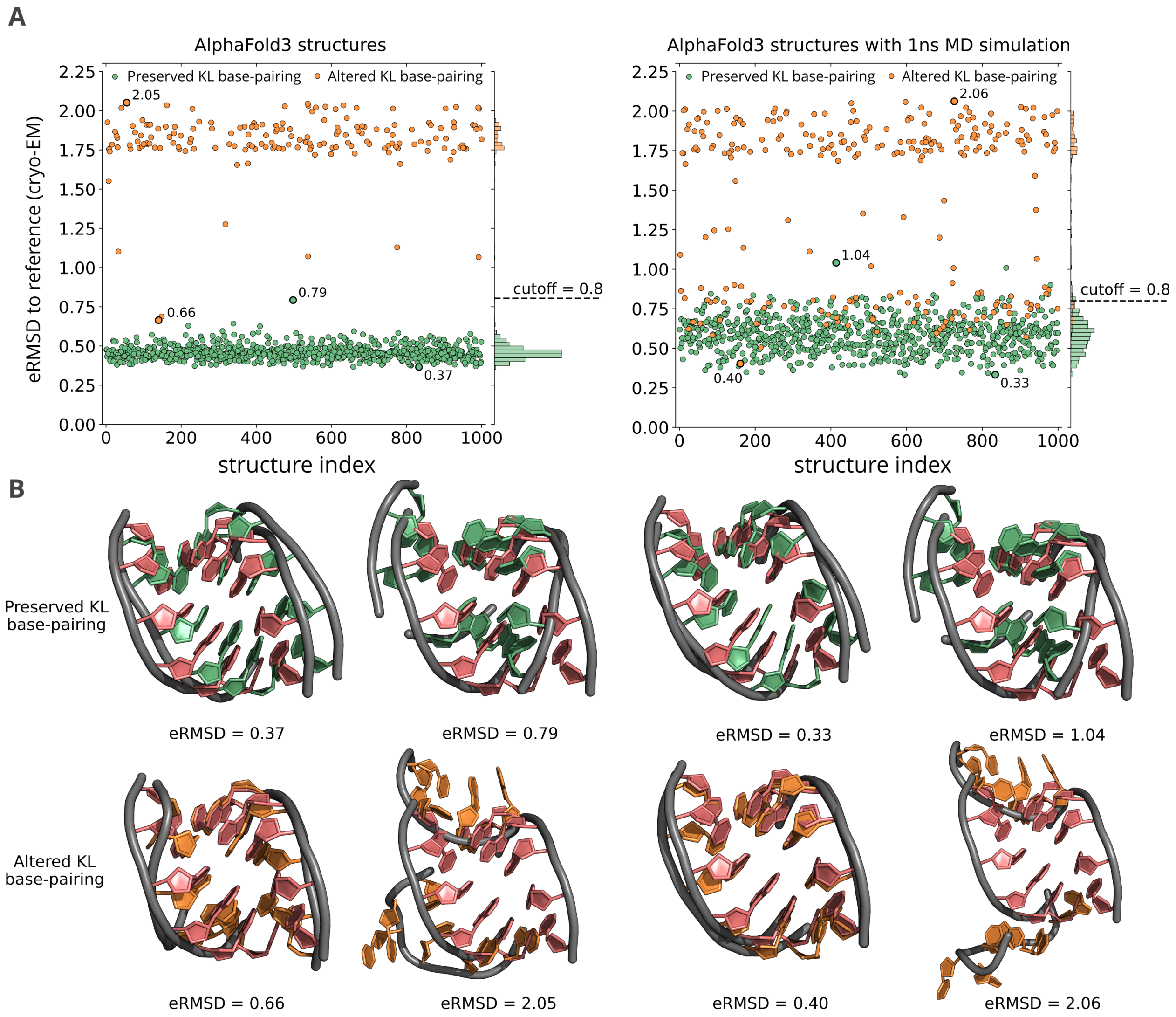

Fig. S9: Correct kissing loop formation and eRMSD values for the AlphaFold3 structure collection before and after 1ns MD simulation. **(A)** eRMSD of all structures from the AlphaFold3 collection before (left) and after (right) 1 ns MD simulation, relative to the reference cryo-EM structure, focusing on the kissing loop region. **(B)** Representative structures classified as correctly forming the kissing loop (with the lowest and highest eRMSD), and incorrectly forming the kissing loop (also with the lowest and highest eRMSD), both before and after MD simulation.

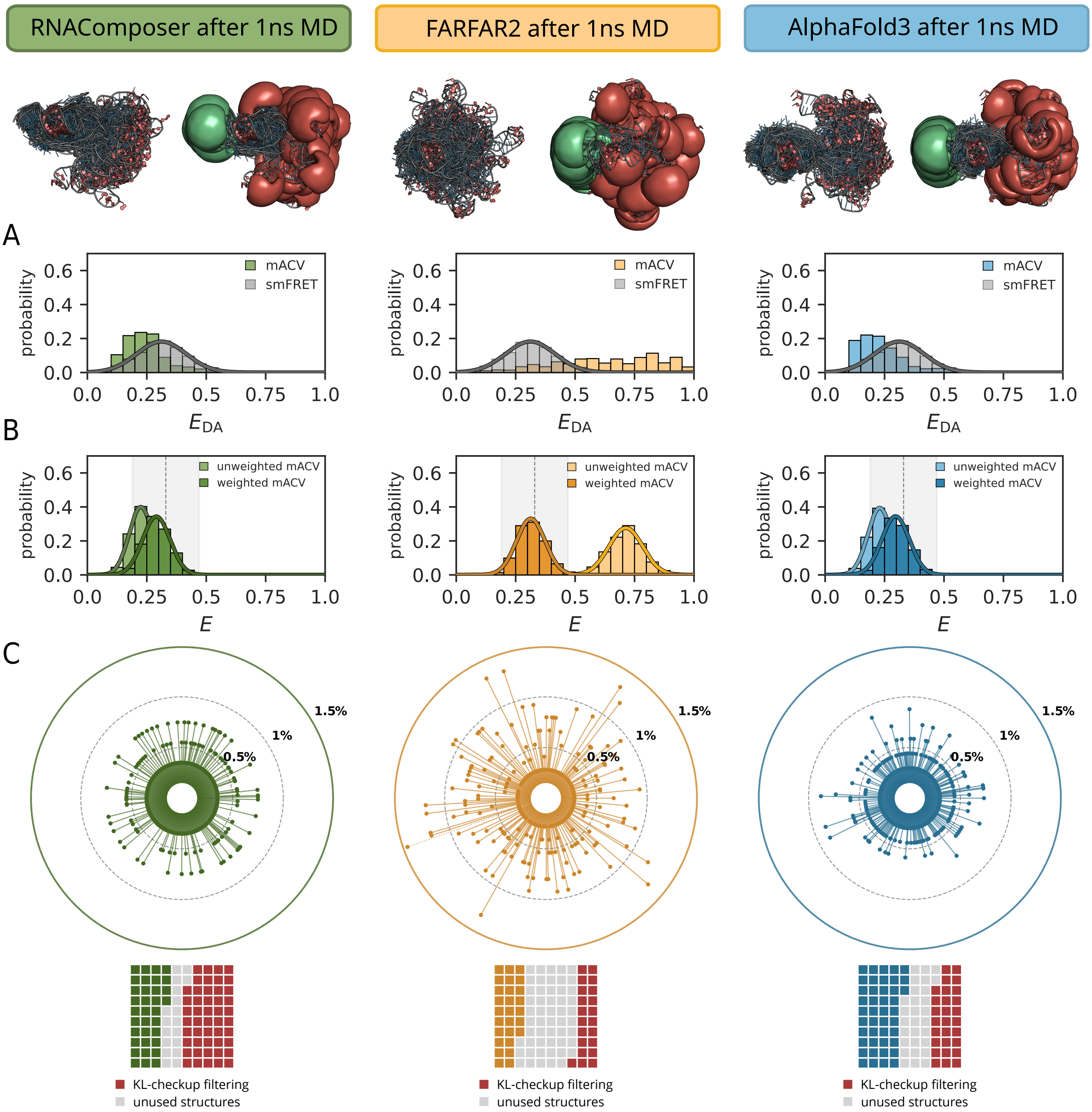

Fig. S10: FRET distributions and structural composition of model collections after 1 ns MD simulation. **(A)** FRET distributions obtained from all methods without photon sampling. **(B)** FRET distributions calculated with photon statistics using FRETraj (19). "Unweighted" refers to uniform structure contributions during burst calculation; "weighted" reflects sampling based on experimental smFRET probabilities. **(C)** Structure contributions of each tool of the weighted approach. A more uniform distribution of structures indicate a greater structural diversity and therefore a larger number of models representing the unbound state. The waffle chart illustrates the reduction in structure numbers after filtering for validated kissing loops (red fractions) and the final number of structures used in the weighting analysis (colored fractions).

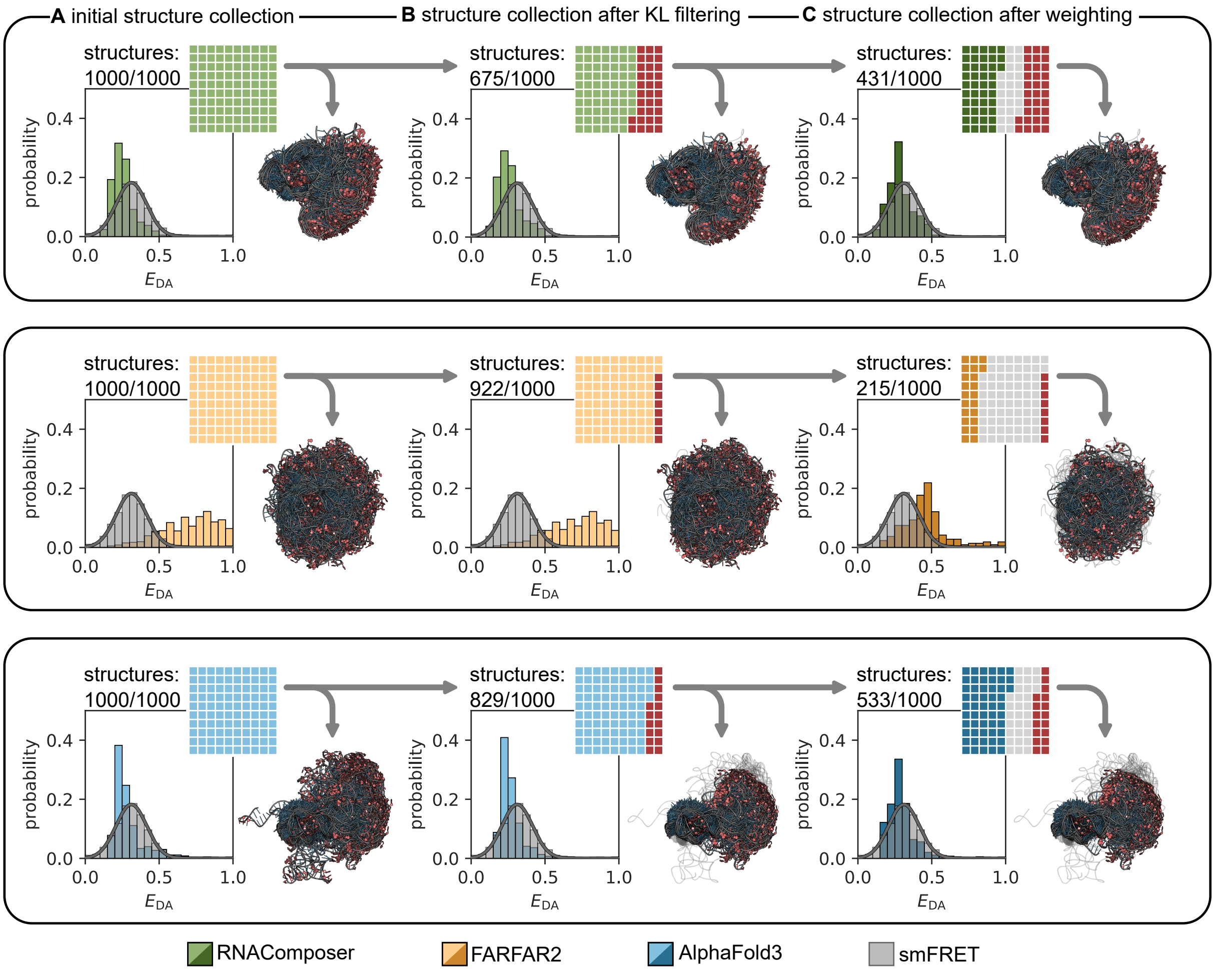

Fig. S11: Comparison of initial, KL-filtered, and weighted E_{DA} distributions and their corresponding structure collections. For each RNA 3D prediction tool, histograms show the calculated E_{DA} distribution of the respective structure collections overlaid with the experimental smFRET E_{DA} distribution. **(A)** Initial structure collections containing 1000 structures. **(B)** Structure collections after kissing-loop (KL) filtering. **(C)** Final structure collections after FRET-guided weighting. The structures underlying each E_{DA} distribution are shown next to the corresponding plot.

Supplementary Tables

Table S1: FRET correction factors for spectral crosstalk including bleed-through (bt) and direct excitation (dE), as well as the γ-correction factor with detection efficiency η and quantum yield Q.

Category	Correction factor	Donor	Acceptor
Bleed-through	bt	9.1%	0.3%
Direct excitation	dE	0.2%	4.2%
Detection efficiency	η	$\eta_A/\eta_D = 2.72$	
Quantum yield	Q	0.46	0.35

Table S2: Fit parameters of the fluorescence lifetime for donor-only (Cy3) and donor-acceptor labeled (Cy5) samples (20).

Parameter	Symbol	Donor (Cy3)	Acceptor (Cy5)
Lifetime component 1	τ_1 (ns)	0.184 ± 0.013	1.008 ± 0.015
Lifetime component 2	τ_2 (ns)	0.859 ± 0.058	1.773 ± 0.017
Lifetime component 3	τ_3 (ns)	1.976 ± 0.045	–
Weighting factor 1	a_1	0.77	0.65
Weighting factor 2	a_2	0.18	0.35
Weighting factor 3	a_3	0.05	–
Average lifetime	τ_{av} (ns)	0.925 ± 0.042	1.3823 ± 0.0008

Table S3: Fit parameters from time-resolved fluorescence anisotropy fits using the local-global rotation-in-a-cone model for donor-only (Cy3) and donor-acceptor labeled (Cy5) samples (20).

Parameter	Symbol	Donor (Cy3)	Acceptor (Cy5)
Fundamental anisotropy	r_0	0.356 ± 0.019	0.349 ± 0.013
Residual anisotropy	r_∞	0.2405 ± 0.0067	0.1152 ± 0.0099
Correlation time local component	$\tau_{r,\mathrm{loc}}$ (ns)	0.329 ± 0.096	0.94 ± 0.12
Correlation time global component	$\tau_{r,\mathrm{glob}}$ (ns)	7.54 ± 0.32	15.7 ± 2.9
Free fraction	w_{free} (%)	32.38	66.99
Stacked fraction	w_{stacked} (%)	67.62	33.01
Hydrodynamic radius (local)	$R_h^{(\tau)}$ (nm)	0.688 ± 0.091	0.977 ± 0.073
Hydrodynamic radius (global)	$R_h^{(D)}$ (nm)	1.955 ± 0.095	2.49 ± 0.24

Table S4: Overview of structure loss due to kissing loop filtering and entanglements found in the remaining structures.

	Initial collection	After KL check	w/o entanglements
RNAComposer	1,000	675	338
RNAComposer (after 1ns MD)	1,000	527	269
FARFAR2	1,000	935	139
FARFAR2 (after 1ns MD)	1,000	803	96
AlphaFold3	1,000	829	17
AlphaFold3 (after 1ns MD)	1,000	716	52

Table S4 highlights the importance of filtering implausible artificially generated 3D structures across the three RNA 3D prediction tools. Although RNAComposer loses a significant fraction of its models due to eRMSD and Watson–Crick base-pairing filters, only about half of the remaining structures exhibit entanglements. In contrast, FARFAR2 and AlphaFold3 retain most structures after our initial filtering, but nearly all are removed when additionally filtering for entanglements detected by RNAspider (21).

References

1. Gerhardy, S., Oborská-Oplová, M., Gillet, L., *et al.* (2021) Puf6 primes 60S pre-ribosome nuclear export at low temperature. *Nat Commun*, **12**, 4696. DOI: 10.1038/s41467-021-24964-2
2. Wahl, M., Röhlicke, T., Rahn, H.-J., *et al.* (2013) Integrated multichannel photon timing instrument with very short dead time and high throughput. *Rev Sci Instrum*, **84**, 043102. DOI: 10.1063/1.4795828
3. Kapanidis, A.N., Lee, N.K., Laurence, T.A., *et al.* (2004) Fluorescence-aided molecule sorting: analysis of structure and interactions by alternating-laser excitation of single molecules. *Proc Natl Acad Sci USA*, **101**, 8936–8941. DOI: 10.1073/pnas.0401690101
4. Hellenkamp, B., Schmid, S., Doroshenko, O., *et al.* (2018) Precision and accuracy of single-molecule FRET measurements-a multi-laboratory benchmark study. *Nat Methods*, **15**, 669–676. DOI: 10.1038/s41592-018-0085-0
5. Lee, N.K., Kapanidis, A.N., Wang, Y., *et al.* (2005) Accurate FRET measurements within single diffusing biomolecules using alternating-laser excitation. *Biophys J*, **88**, 2939–2953. DOI: 10.1529/biophysj.104.054114
6. McCann, J.J., Choi, U.B., Zheng, L., *et al.* (Aug. 4, 2010) Optimizing methods to recover absolute FRET efficiency from immobilized single molecules. *Biophys J*, **99**, 961–970. DOI: 10.1016/j.bpj.2010.04.063
7. Sarzynska, J., Popenda, M., Antczak, M., *et al.* (2023) RNA tertiary structure prediction using RNAComposer in CASP15. *Proteins*, **91**, 1790–1799. DOI: 10.1002/prot.26578
8. Watkins, A.M., Rangan, R., Das, R. (2020) FARFAR2: Improved DE Novo Rosetta prediction of complex global RNA folds. *Structure*, **28**, 963–976.e6. DOI: 10.1016/j.str.2020.05.011
9. Steffen, F.D., Cunha, R.A., Sigel, R.K.O., *et al.* (2024) FRET-guided modeling of nucleic acids. *Nucleic Acids Res*, **52**, e59. DOI: 10.1093/nar/gkae496
10. Abramson, J., Adler, J., Dunger, J., *et al.* (2024) Accurate structure prediction of biomolecular interactions with AlphaFold 3. *Nature*, **630**, 493–500. DOI: 10.1038/s41586-024-07487-w
11. Abraham, M.J., Murtola, T., Schulz, R., *et al.* (2015) GROMACS: High performance molecular simulations through multi-level parallelism from laptops to supercomputers. *SoftwareX*, **1-2**, 19–25. DOI: 10.1016/j.softx.2015.06.001
12. Cornell, W.D., Cieplak, P., Bayly, C.I., *et al.* (1995) A second generation force field for the simulation of proteins, nucleic acids, and organic molecules. *J Am Chem Soc*, **117**, 5179–5197. DOI: 10.1021/ja00124a002
13. Pérez, A., Luque, F.J., Orozco, M. (2007) Dynamics of B-DNA on the microsecond time scale. *J Am Chem Soc*, **129**, 14739–14745. DOI: 10.1021/ja0753546
14. Banáš, P., Hollas, D., Zgarbová, M., *et al.* (2010) Performance of molecular mechanics force fields for RNA simulations: Stability of UUCG and GNRA hairpins. *J Chem Theory Comput*, **6**, 3836–3849. DOI: 10.1021/ct100481h
15. Zgarbová, M., Otyepka, M., Sponer, J., *et al.* (2011) Refinement of the Cornell et al. Nucleic acids force field based on reference quantum chemical calculations of glycosidic torsion profiles. *J Chem Theory Comput*, **7**, 2886–2902. DOI: 10.1021/ct200162x

16. Abascal, J.L.F., Sanz, E., García Fernández, R., *et al.* (2005) A potential model for the study of ices and amorphous water: TIP4P/Ice. *J Chem Phys*, **122**, 234511. DOI: 10.1063/1.1931662

17. Wu, S., Tutuncuoglu, B., Yan, K., *et al.* (2016) Diverse roles of assembly factors revealed by structures of late nuclear pre-60S ribosomes. *Nature*, **534**, 133–137. DOI: 10.1038/nature17942

18. Schrödinger, LLC. "The PyMOL Molecular Graphics System, Version 2.5.10". Schrödinger, LLC. Nov. 2015.

19. Steffen, F.D., Sigel, R.K.O., Börner, R. (2021) FRETraj: integrating single-molecule spectroscopy with molecular dynamics. *Bioinformatics*, **37**, 3953–3955. DOI: 10.1093/bioinformatics/btab615

20. Steffen, F.D., Sigel, R.K.O., Börner, R. (2016) An atomistic view on carbocyanine photophysics in the realm of RNA. *Phys Chem Chem Phys*, **18**, 29045–29055. DOI: 10.1039/C6CP04277E

21. Luwanski, K., Hlushchenko, V., Popenda, M., *et al.* (2022) RNAspider: a webserver to analyze entanglements in RNA 3D structures. *Nucleic Acids Res*, **50**, W663–W669. DOI: 10.1093/nar/gkac218
